# Chemical Diversity and Classification of Secondary Metabolites in Nine Bryophyte Species

**DOI:** 10.3390/metabo9100222

**Published:** 2019-10-11

**Authors:** Kristian Peters, Hendrik Treutler, Stefanie Döll, Alida S. D. Kindt, Thomas Hankemeier, Steffen Neumann

**Affiliations:** 1 Leibniz Institute of Plant Biochemistry, Bioinformatics and Scientific Data, Weinberg 3, 06120 Halle (Saale), Germany; Hendrik.Treutler@ipb-halle.de (H.T.); Stefanie.Doell@ipb-halle.de (S.D.); sneumann@ipb-halle.de (S.N.); 2 Division of Analytical Biosciences, Leiden Academic Centre for Drug Research (LACDR), Leiden University, 2333 CC Leiden, The Netherlands; a.s.d.kindt@lacdr.leidenuniv.nl; 3 Division of Systems Biomedicine and Pharmacology, Leiden Academic Centre for Drug Research (LACDR), Leiden University, 2333 CC Leiden, The Netherlands; hankemeier@lacdr.leidenuniv.nl; 4 German Centre for Integrative Biodiversity Research (iDiv) Halle-Jena- Leipzig, Deutscher Platz 5e, 04103 Leipzig, Germany

**Keywords:** ecometabolomics, mosses, bryophytes, chemodiversity, biodiversity, compound classes, classification, chemical ecology, data-dependent acquisition, clustering, massbank

## Abstract

The central aim in ecometabolomics and chemical ecology is to pinpoint chemical features that explain molecular functioning. The greatest challenge is the identification of compounds due to the lack of constitutive reference spectra, the large number of completely unknown compounds, and bioinformatic methods to analyze the big data. In this study we present an interdisciplinary methodological framework that extends ultra-performance liquid chromatography coupled to electrospray ionization quadrupole time-of-flight mass spectrometry (UPLC/ESI-QTOF-MS) with data-dependent acquisition (DDA-MS) and the automated *in silico* classification of fragment peaks into compound classes. We synthesize findings from a prior study that explored the influence of seasonal variations on the chemodiversity of secondary metabolites in nine bryophyte species. Here we reuse and extend the representative dataset with DDA-MS data. Hierarchical clustering, heatmaps, dbRDA, and ANOVA with post-hoc Tukey HSD were used to determine relationships of the study factors species, seasons, and ecological characteristics. The tested bryophytes showed species-specific metabolic responses to seasonal variations (50% vs. 5% of explained variation). *Marchantia polymorpha*, *Plagiomnium undulatum*, and *Polytrichum strictum* were biochemically most diverse and unique. Flavonoids and sesquiterpenoids were upregulated in all bryophytes in the growing seasons. We identified ecological functioning of compound classes indicating light protection (flavonoids), biotic and pathogen interactions (sesquiterpenoids, flavonoids), low temperature and desiccation tolerance (glycosides, sesquiterpenoids, anthocyanins, lactones), and moss growth supporting anatomic structures (few methoxyphenols and cinnamic acids as part of proto-lignin constituents). The reusable bioinformatic framework of this study can differentiate species based on automated compound classification. Our study allows detailed insights into the ecological roles of biochemical constituents of bryophytes with regard to seasonal variations. We demonstrate that compound classification can be improved with adding constitutive reference spectra to existing spectral libraries. We also show that generalization on compound classes improves our understanding of molecular ecological functioning and can be used to generate new research hypotheses.

## 1. Introduction

Bryophytes or ‘mosses’ are considered to be the oldest group of terrestrial plants [1]. They are classified into the three major groups liverworts (‘hepatics’, Marchantiophyta), mosses *s. str.* (‘musci’, Bryophyta) and hornworts (Anthocerophyta) [2,3,4]. As of today, there are approx. 24,000 bryophyte species known [5]. Unlike vascular plants, bryophytes lack well developed cell structures that protect them from herbivores, pathogens, and environmental exposures. As a result, they have evolved a unique diversity of bioactive compounds as part of their survival strategy [6]. Some bioactive compounds may have also evolved in early stages of land plant evolution and are thus present in both bryophytes and vascular plants.

Bryophytes are small and morphologically inconspicuous plants. Yet, their biochemistry is relatively unstudied despite the fact that some species have been widely used in traditional medicine of indigenous people around the world, especially in China [7]. While terpenoids constitute the largest group of bioactive compounds, bryophytes also produce a variety of glycosides, flavonoids, and other aromatic compounds [8,9]. Restricted to liverworts are cellular oil bodies that contain many unique compounds such as cyclic neolignans ((bis)-bibenzyls), diverse sesquiterpene lactone derivatives, other terpenoids, and fatty acids (see Table 1 for an overview on most common compound classes in bryophytes). Hence the name “oil bodies” as they appear under the microscope as little droplets of oil [10]. Many of these compounds are considered to be bioactive [5,6].

Bryophytes produce a variety of metabolic compounds as defense against mechanical damage, environmental changes, and pathogens [9,11,12]. Metabolites play also a role in interactions of bryophytes with other organisms. Here the research field of ecometabolomics has the unique potential to relate specific compounds to ecological functioning [13]. For example, it has been shown that the species *Dicranum scoparium* produces acetylic oxylipins as a defense against herbivorous slugs [14,15] and that some bryophytes can inhibit the germination of vascular plant seeds [16,17]. Ecometabolomics is also a useful tool to elucidate the importance of bryophytes in carbon and nitrogen cycling [18,19] and the biochemical role of bryophytes within biological soil crusts [20,21]. Some desiccation-tolerant bryophytes have been described as “resurrection plants” [22]. While it is known that many bryophytes have developed structures such as hyaline hairs or convoluting leaves to reduce evaporation and exposure to radiation, ecometabolomics could help to unravel the chemical constituents in the cells of bryophytes that are associated with water stress [23]. Ecometabolomics can further be used to explore the ecological or environmental circumstances under which bryophytes produce certain natural products, e.g., those that have medicinal value [5,24]. Thus, in ecometabolomics the goal is to find molecular mechanisms that allow the ecological interpretation at more coarse spatiotemporal scales [13]. In a top-down approach, sets of biomarkers are sought that explain ecological functioning or organismal interactions [25]. These biomarkers can be constituted by any kind of natural products—substances, secondary metabolites, or even compound classes [26].

Biochemical fingerprints can be generated from the presence or absence of compounds and their relative intensity can be used to describe chemotaxonomic properties that discriminate different species, ecotypes, or phylogenetic relationships [27,28]. High-throughput techniques like GC/MS or LC/MS offer unique possibilities but were rarely applied to bryophytes with regard to chemosystematics [29]. Liquid chromatography coupled to mass-spectrometry and data-dependent acquisition (DDA-MS) is a relatively fast and inexpensive analytical method that subjects the most abundant MS1 peaks to a second stage of MS2 fragmentation in a defined series of scans before the cycle starts again [30]. The method potentially allows the elucidation of the most abundant natural products in biological species and is a useful analytical tool to assess the chemodiversity between samples of different biological species [31]. A full scan automatically selects the precursor ions for isolation according to intensity thresholds and priority settings. Precursors are then subjected to collision induced dissociation CID) with variable collision energies. With DDA-MS, usually several hundred high quality MS2 fragment spectra can be acquired per analytical run.

In this study, we explore the diversity of compound classes of secondary metabolites of nine different bryophytes species with regard to seasonal variations. Our preceding study and many other ecometabolomics studies have performed metabolite fingerprinting and LC/MS at MS1 level to find biochemical patterns that explain ecological relationships [32,33]. We here extend the methodology towards a more mechanistic characterization by the automatic *in silico* identification of compound classes using additional DDA-MS. As bryophytes and other non-model species are biochemically not well characterized they likely contain novel compounds that have never been measured before (also called “unknown unknowns” and often referred to as compounds as part of the “dark matter”) [34,35]. It is a very complex and time-consuming process to annotate and characterize the chemical structures for these unknown compounds [26] because there are no analytical standards available and very sparse spectral annotations are available in reference libraries. The only options currently available require either manual analytical characterization which often involves a complete de novo structure elucidation with additional methods such as NMR [34], or in silico approaches that predict chemical structures based on molecular fingerprints or fragmentation patterns via computational methods [36,37,38].

To overcome these limitations, we explore an additional *in silico* method that performs automatic classification of compounds into compound classes based on recognized ontologies [38,39,40]. To this end, we annotated unknown MS2 fragment spectra with terms from the ChemOnt ontology using ClassyFire [40]. ChemOnt is a purely molecular structure-based chemical taxonomy that comprises structures and structural features to assign known compounds to a taxonomy using more than 4800 categories defined by computable structural rules and a consensus-based nomenclature [39,40]. We use these ontological categories to train a classifier with reference fragment spectra of known compounds based on the MassBank library [41,42]. We use the resulting classifier to assign unknown fragment spectra to compound classes which were acquired for the different bryophyte species in our analytical pipeline.

Our methodology improves subsequent ecometabolomics analyses as it allows a better understanding of chemodiversity and deeper insight into biochemical processes than metabolite fingerprinting alone. By organizing chemical entities into classes [43], they can be used to observe ecological or diversity relationships at molecular levels and to assign ecological functioning to compound classes.

The goals of the study are (1) to present a novel processing pipeline that combines DDA-MS with the automated classification of fragment peaks of the most abundant chemical entities into compound classes, and (2) to present an integrative bioinformatic framework based on a representative dataset to provide a reusable bioinformatic toolset to improve subsequent ecometabolomics studies and to generate new research hypotheses.

## 2. Materials and Methods

For experimental design and sampling we refer to our previous study [33]. Briefly, the nine moss species *Brachythecium rutabulum* (Hedw.) Schimp., *Calliergonella cuspidata* (Hedw.) Loeske, *Hypnum cupressiforme* Hedw., and *Rhytidiadelphus squarrosus* (Hedw.) Warnst. (group pleurocarpous mosses), *Fissidens taxifolius* Hedw., *Grimmia pulvinata* (Hedw.) Sm., *Plagiomnium undulatum* (Hedw.) T.J. Kop., *Polytrichum strictum* Menzies ex Brid. (group acrocarpous mosses), and *Marchantia polymorpha* L. (group liverworts) were sampled in a natural environment in the Botanical Gardens of the Martin Luther University Halle-Wittenberg, Germany in the seasons summer (2016/08/08), autumn (2016/11/09), winter (2017/01/27) and spring (2017/05/11).

Based on aliquots of methanolic extracts produced in [32], ultra-performance liquid chromatography coupled to electrospray ionization quadrupole time-of-flight mass spectrometry (UPLC/ESI-QTOF-MS) was performed acquiring collision induced dissociation mass spectra (CID) using a MicroTOF–Q hybrid quadrupole time-of-flight mass spectrometer equipped with an Apollo II electrospray ion source (Bruker Daltonics, Billerica, MA, USA).

Chromatographic separations were performed at 40 °C on an Acquity UPLC system (Waters, Milford, MA, USA) equipped with an HSS T3 column (100 × 1 mm, particle size 1.8 µm; Waters, Milford, MA, USA). A binary gradient was applied at a flow rate of 150 µL/min^-1^ at 0 to 1 min, isocratic 95% A (water: formic acid: 99.9:0.1 (*v/v*)), 5% B (acetonitrile: formic acid: 99.9:0.1 (*v/v*)); 1 to 18 min, linear from 5% to 95% B; 18 to 20 min, isocratic 95% B. The injection volume was 3.1 µL (full loop injection).

Metabolite profiles were acquired with the following MS instrument settings: ionization mode: positive, scan range: 50–1000 m/z, nebulizer and dry gas nitrogen at 1.6 bar and 6 L/min, source temperature: 190 °C, capillary voltage: 5000 V, end plate offset: −500 V, ion optics: funnel 1 RF 200 Vpp, funnel 2 RF 200 Vpp, in-source CID energy: 0 eV, hexapole RF 100 Vpp, quadrupole ion energy 3 eV, collision gas: argon, isolation mass: 100 m/z, collision energy: 3 eV, collision cell RF 200 Vpp, transfer time: 70 µs, pre pulse storage time: 5 µs, spectra rate: 3 Hz.

MS2 fragment spectra of most abundant peaks were acquired in data-dependent acquisition mode (DDA-MS or sometimes also referred to as ‘Auto-MS-MS’) with the following instrument settings: CID mode, number of precursors: 3, intensity threshold: 600, precursor background subtraction enabled, active exclusion enabled after 3 spectra, release after 18 s, priority list for isolation at 300 m/z: 1z, 2z, 3z, at 500 m/z: 1z, 2z, 3z, at 1000 m/z: 1z, 2z, 3z, isolation width: 3–10 m/z (isolation width at 300 Da: 3 m/z, at 500 Da: 8 m/z, at 1000 Da: 10 m/z), collision energy: 15–70 eV (collision energy at 300 m/z for charge states 1z, 2z, 3z: 15, 20, 25 eV, at 500 m/z for charge states 1z, 2z, 3z: 30, 35, 45 eV, at 1000 m/z for charge states 1z, 2z, 3z: 40, 50, 70 eV).

Detection of chromatographic peaks was performed in R 3.5.3 with the package XCMS 1.52.0 and CAMERA 1.33.3 [44,45] using the parameters described in [33]. The additional packages parallel, foreach, doMC, RColorBrewer, multtest, MSnbase, mixOmics, vegan, multcomp, and Hmisc were used for statistical analyses.

Quality control was employed with the mzQuality workflow for MS data (Further information is available at http://www.mzQuality.nl) Here, the relative standard deviation (RSD) was calculated for the eight internal standards injected into 18 quality control (QC) samples. Furthermore, a plot depicting the batch design and the intensity measured in 22 metabolites in all measured samples was produced (Supplemental Figure S7).

Metabolite richness and diversity indices were calculated by determining the unique chemical entities (those features that are only present in one of the study factors (“species” or “seasons”) but not the others), by calculating the Shannon diversity index *H’*, and by determining the peak intensities of chromatographic peaks [33]. To measure the homogeneity of the distribution of metabolite features, the evenness (or equitability) was determined calculating the Pielou’s *J* index [46,47]. To compare the above diversity indices, boxplots were generated for the two study factors “species” and “seasons”. To test individual factor levels for differences, the Tukey HSD was performed post-hoc. Letters representing the groups were placed in the upper part of the boxplots. In the following, we use the term ‘significant’ when *p*-values are below a threshold of 0.05.

MS2 spectra were extracted from the mzML and converted to NIST MSP format (https://www.nist.gov/sites/default/files/documents/srd/NIST1a11Ver2-0Man.pdf). Fragment spectra similar in precursor mass (mz difference ≤ 0.01 Da) and retention time (Δrt error ≤ 5 s) were merged in each sample. Only compounds with a mass between 50 and 1000 Da and a retention time between 10 and 1020 s were evaluated. Compound classes of all the grouped MS2 fragment spectra were determined using classifiers which were created on a training set of ~57,000 MS2 fragment spectra with known structures from MassBank (René Meier, personal communication) [41,48]. Prior to this study, MassBank have not contained spectra of the group of neolignans (bis-bibenzyls) and sesquiterpenoids have been underrepresented. We thus complemented the library with manually curated spectra of 23 lignans and 27 sesquiterpenoids from identified compounds of *Marchantia polymorpha*. We further annotated these spectra with terms from the ChemOnt ontology using ClassyFire [39,40].

Classes from the ChemOnt ontology (as listed in Table 1) were used to train a classifier for each compound class determined in the bryophyte samples [49]. Spectra were grouped into a foreground data set containing spectra from compounds which are member of the compound class and a background data set containing the remaining spectra. Only those fragment peaks above an abundance threshold of 100 were used. A simple classifier was trained with the foreground and background data set. In the prediction step, the membership of each unknown spectrum was predicted for each compound class. A *p*-value based on the score distribution of the background data set was calculated from the training step. Only predictions with a *p*-value ≤ 0.05 were kept.

As the foreground and background data sets had an imbalanced class ratio (positives are substantially less abundant than negatives), we assessed the correctness of the classifier by calculating the area under the precision–recall curve (AUC-PR) and the true positive rate for a fixed false negative rate of 5% (TPR-FNR) [50]. We calculated the AUC-PR as a single number to evaluate the performance of our binary classifier [51]. Precision–recall curves (PR) are increasingly used in machine learning and a useful alternative to the closely related receiver operating characteristic (ROC) curves [52]. PR are considered a more accurate measure with imbalanced datasets as some performance metrics are better represented than with ROC curves [53].

To estimate the probability that our classifier scores are randomly drawn positive entity higher than a randomly drawn negative entity, we use cross-classification by calculating the true positive rate (TPR = sensitivity) on a false negative rate (FNR = specificity) which is a better estimator of performance than a purely TPR with imbalanced data [54]. Similar to a ROC curve, a two-dimensional curve is created with the TPR being the unit in direction of the x-axis and FNR the unit in direction of the y-axis. This curve is parametrized with a threshold of 5%, where formula (1) and formula (2). The resulting TPR-FNR represents the partial area under the two combined curves.

TPR = true positives/positives,(1)

FPR = false negatives/negatives,(2)

To show an overview on the distribution of compound classes, a sunburst plot was created which indicates the membership of all chemical entities and assigns them to all parent compound classes. Heatmaps (using the gplots R package) were generated for the total counts of chemical entities belonging to the respective compound classes and for the study factors species and seasons using Bray–Curtis dissimilarity. Boxplots were generated for compound classes separately which show the quartiles per species and seasons. To assess the relationship to the phylogenetic tree, dendrograms were generated using the R packages ape, pvclust, dendextend, cba, and phangorn. The chemotaxonomic and the phylogenetic tree were compared by calculating the cophenetic correlation value, the Mantel statistic and the Robinson–Foulds metric as described in [33].

To assess the influence of the study factors species and seasons, variation partitioning using distance-based redundancy analysis (dbRDA) was performed (using the vegan R package) [55]. Categorical factors were transformed to presence–absence matrices prior to the ordination. Using the function envfit in the R package vegan, the influences of ecological characteristics (described in [33]) were determined by fitting the ecological characteristics to the ordination of the compound classes matrix. The projections of the ecological characteristics onto vectors have maximum correlation with the corresponding compound entities of the compound classes matrix. The significance of the fitted vectors was assessed using permutation of the ecological characteristics. The goodness of fit statistic is the squared correlation coefficient (r^2^) and empirical *p*-values were calculated for each ecological characteristic (using statistical procedures built into the envfit function).

## 3. Results

Raw metabolite profiles (see also Figure S8 for raw total ion chromatograms) including the MS2 fragment spectra acquired in DDA-MS mode, corresponding data tables, classifiers and meta-data are available as identifier MTBLS709 in the repository MetaboLights [56].

### 3.1. Classification of Compound Classes

**Figure 1:**
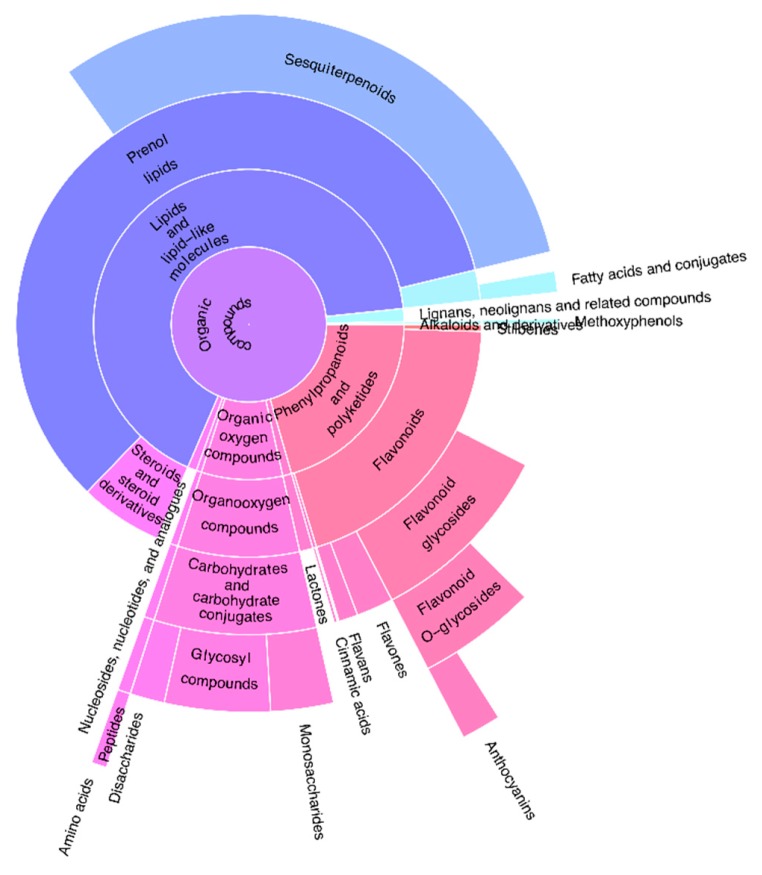
The sunburst plot shows an overview on the classified compound classes in all of the tested bryophyte species. The membership of chemical entities is aggregated and displayed together with all parent compound classes. The width of a compound class in the plot corresponds to the number of chemical entities assigned to this particular class. Our classifier assigned chemical entities to 24 classes out of 35 predefined classes (Table 1). From a total of 10,436 spectra 201 spectra were not matched into any of the classes. Our classifier did not assign fragment spectra to the classes of monoterpenoids, diterpenoids, triterpenoids, benzene and substituted derivatives, benzoic acids, phthalic acid and derivatives, coumarins and derivatives, biflavonoids and polyflavonoids, oligosaccharides, polysaccharides, organic acids and derivatives.

Our classifier assigned chemical entities to 24 compound classes out of 35 predefined classes (listed in Table 1). As shown in Figure 1, the most prevalent compound classes were lipid-like molecules such as prenol lips (including sesquiterpenoids and steroids). Flavonoids and carbohydrates were frequently detected by our setup. We annotated the other bioactive compound classes of methoxyphenols, neolignans, cinnamic acids, lactones, and anthocyanins. Primary metabolites such as saccharides, nucleotides, and amino acids were also determined.

### 3.2. Species-Specific Variations in the Compound Classes

**Figure 2:**
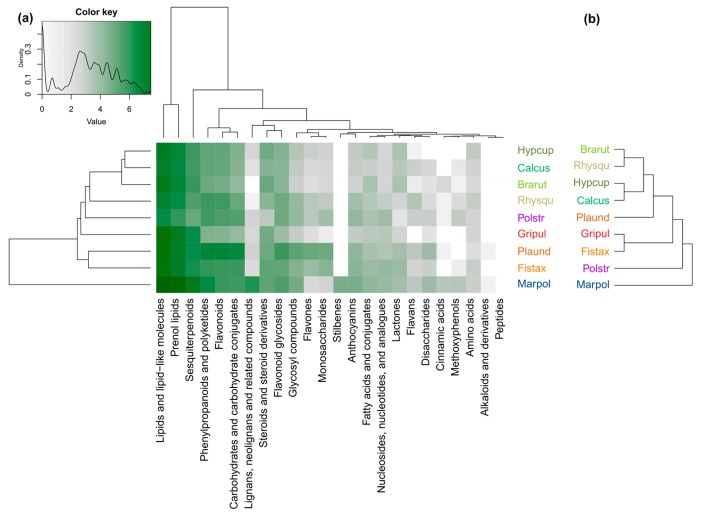
Richness of chemical entities in the different compound classes of the bryophyte species, and chemotaxonomic vs. phylogenetic tree. (**a**) The heatmap shows the richness of compound classes in the bryophyte species. The intensity of the green color corresponds to the number of chemical entities. The color key represents the legend of the heatmap plot and shows the density distribution of counts throughout the range of values which is represented by white (zero counts) and green (counts per species) colors at a logarithmic scale. (**b**) Phylogenetic tree showing the phylogenetic relationships of the bryophyte species. Comparison metrics of the chemotaxonomic tree and the phylogenetic tree: Cophenetic correlation = 0.165; Mantel statistic = 0.16; Robinson–Foulds metric = 0.875.

**Figure 3:**
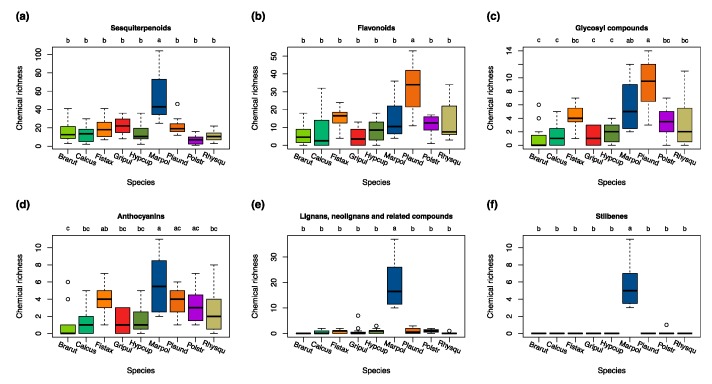
Richness of chemical entities of the different bryophyte species within the chosen chemical classes of (**a**) sesquiterpenoids; (**b**) flavonoids; (**c**) glycosyl compounds; (**d**) anthocyanins; (**e**) lignans; neolignans, and related compounds; and (**f**) stilbenes. Differences among groups (letters on the top of the plot) are based on the Tukey HSD post hoc test on a one-way ANOVA. Different letters show significant differences (*p* < 0.05). *n* = 12 for each species. Red colors represent acrocarpous mosses. Green colors represent pleurocarpous mosses. A blue color represents liverworts.

The high chemical diversity of *Marchantia polymorpha* (as shown in Figure S1a–d and Figure S2a) is mainly the result of the presence of many lipid-like compounds such as sesquiterpenoids (Figure 2a), phenylpropanoids, polyketides (Figure S4h), and flavonoid glycosides (Figure S4o). We detected significantly (*p* < 0.05) more lignans, neolignans, and related compounds than in any other species (Figure 2a, Figure 3e). Stilbenes were present only in *M. polymorpha* (Figure 3f). The relatively high chemodiversity of *Plagiomnium undulatum* was due to large numbers of flavonoids, flavones, and monosaccharides and glycosyl compounds in comparison to the other species (including phenylpropanoids and polyketides and carbohydrates and carbohydrate conjugates as parent classes) (Figure 2a, Figure 3b,c, Figure S4). Generally, a low diversity was observed in profiles of *Polytrichum strictum* (Figure S1b, Figure S2a). This was significant (*p* < 0.05) for steroids and steroid derivatives (Figure S4c), sesquiterpenoids (Figure 3a), and pronounced for lactones (Figure S4g). However, we detected many unique chemical entities regardless of class that were not present in any of the other species (Figure S1a). *Fissidens taxifolius* had a larger richness in the classes of phenylpropanoids and polyketides, flavonoids, flavones, flavonoid glycosides, anthocyanins, carbohydrates and conjugates, glycosyl compounds, and monosaccharides when compared to other acrocarpous and pleurocarpous mosses (Figure S3b–d, Figure S4h,l,m,o–r). The slightly larger diversity of acrocarpous mosses over the group pleurocarpous mosses (Figure S1b, Figure S2a, Figure S4) was mostly due to lower chemical richness in the classes of prenol lipids and carbohydrates (including glycosylic compounds) in pleurocarpous mosses (Figure 2a).

The liverwort *M. polymorpha* was the most basal species in both the chemotaxonomic and the phylogenetic tree (Figure 2a,b). When compared to the phylogenetic tree, *P. strictum* had a different position which was mainly the result of an overall low chemical richness and the presence of many unique chemical entities, especially methoxyphenols (Figure 2a,b, Figure S4k). The different position of *P. undulatum* and *F. taxifolius* within the group of acrocarpous mosses was mainly due to the presence of many characteristic phenylpropanoids and polyketides (including flavonoids and flavones), lactones, anthocyanins, and carbohydrates (including monosaccharides and glycosyl compounds) (Figure 2a,b, Figure 3b–d, Figure S4). The pleurocarpous mosses *H. cupressiforme*, *C. cuspidata*, and *B. rutabulum* had a rather homogeneous richness in the compound classes, except for carbohydrates and its conjugates and anthocyanins (Figure 2a,b, Figure 3d, Figure S4q–t).

### 3.3. Seasonal Variations in the Compound Classes

**Figure 4:**
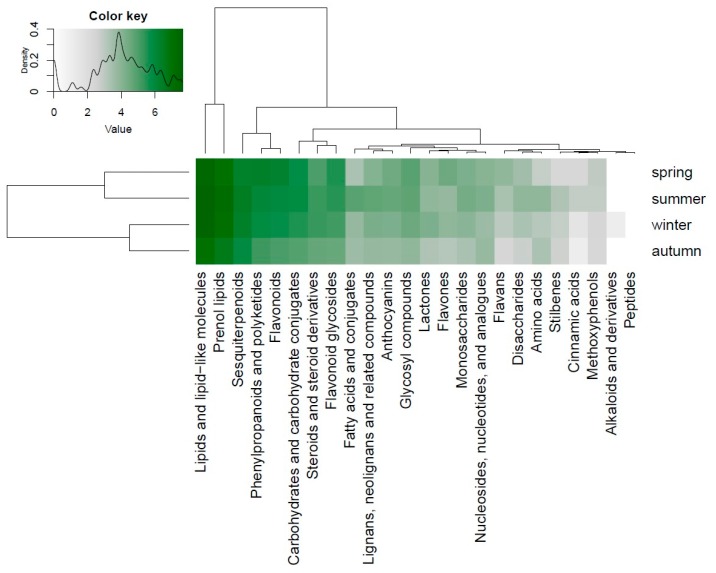
The heatmap shows the richness in the compound classes and the relationship to the study factor seasons. The intensity of the green color corresponds to the number of chemical entities. The color key represents the legend of the heatmap plot and shows the density distribution of counts throughout the range of values which is represented by white (no counts) and green (many counts) colors at a logarithmic scale.

**Figure 5:**
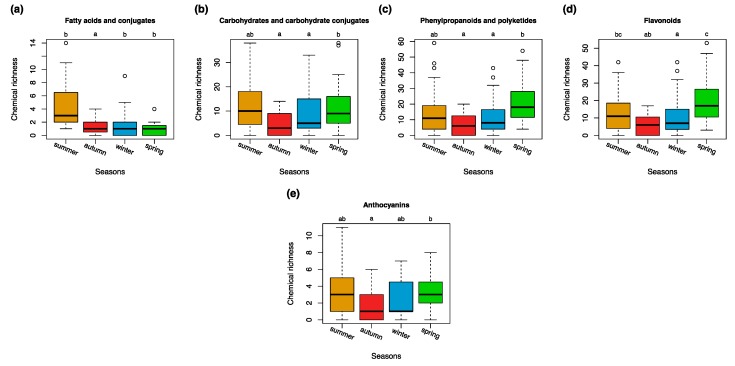
Seasonal variation in the richness of chemical entities (features) within the chosen chemical classes of (**a**) fatty acids and conjugates, (**b**) carbohydrates and carbohydrate conjugates, (**c**) phenylpropanoids and polyketides, (**d**) flavonoids, and (**e**) anthocyanins. Differences among groups (letters on the top of the plot) are based on the Tukey HSD post hoc test on a one-way ANOVA. Different letters show significant differences (*p* < 0.05). *n* = 27 for each season.

All tested bryophytes produced more compounds in spring and summer than in autumn. This effect was significant (*p* < 0.05) for fatty acids and conjugates, carbohydrates, and glycosyls, phenylpropanoids and polyketides, flavonoids and anthocyanins (Figure 4, Figure 5, Figure S5). The classes of steroids and steroid derivatives, lactones and sesquiterpenoids were enriched in the winter season (Figure S5c,g,e).

### 3.4. Seasonal Variations in Compound Classes in Different Bryophyte Species

Variation partitioning showed that the study factor species explained 50% and the factor seasons accounted for 5% of the variations in the compound classes matrix (Figure S3b). Thus, species responded very specific to seasonal variations. The species *M. polymorpha*, *P. undulatum*, and *P. strictum* showed the largest seasonal variations in compound classes (Table S1). Acrocarpous species were more variable when compared to pleurocarpous species. Full material is available in the supplement in Figure S6.

The chemical richness of many compound classes was increased in spring and summer and reduced in the autumn season for most of the species (see Figure S6 for full details). Some compound classes were enriched in winter for acrocarpous species and the pleurocarpous *B. rutabulum* (Figure S6).

## 4. Discussion

Formerly, the determination of compound classes required the use of elaborate analytical methods which often take biochemists months of work by the combination of chromatographic separation techniques and different extraction methods to characterize the different sets of compound classes [12,26,29,57,58,59]. Our proposed automatic framework combines a data dependent acquisition mass spectrometry (DDA-MS) analysis of methanolic extracts with *in silico* compound classification. The analytical MS2 pipeline is able to capture most semi-polar secondary metabolites. Our compound classification method offers to acquire the data more quickly by avoiding the complex biochemical preparation procedures. In the following, we discuss how such a framework will augment the generalization and identification of molecular patterns in ecological contexts.

Vascular plants show strong differences in metabolic responses to above- and below-ground pathogens and environmental factors [60]. To our knowledge, it has never been tested whether bryophytes differ in their above- and below-ground metabolic responses to pathogens and environmental fluctuations; and whether different sets of compounds and compound classes can be associated [61]. In our experiment, we sampled the gametophytes of bryophytes without rhizoids and without reproductive parts. As bryophytes are closely associated with the substrate due to their stature, it is likely that even in metabolite profiles of above-ground parts many secondary metabolites are present that indicate ecological interactions that occur below-ground [62,63,64]. Future studies should also measure soil-related properties such as nutrient levels (C,N) and may even record soil microbiota by a metagenomics approach [65,66]. This wealth of information is perfectly suited to not just characterize several organismal interactions (such as the responses of bryophytes to pathogens, herbivores and microbials) but also to explore the metabolic differences and life strategies of bryophytes of type pioneers vs. stayers [67] and to identify metabolites that are associated with C-, S-, or R-selection strategies [68].

The results of our study agree with other studies that liverworts produce more compounds than mosses; and that this is not due to the fact that liverworts are biochemically better studied than mosses [5,8]. However, our study additionally showed great variation in chemodiversity. The most abundant metabolites are related to species-specific effects that are produced to sustain the homeostasis of the metabolism [69]. We found an estimate of 1/3 of compound numbers that are directly related to environmental changes such as seasonal variations but they only make approx. 1/10 of the overall peak intensity. This suggests that many metabolic compounds are highly specific and are only produced when needed, e.g., upon environmental fluctuation. This explains why many metabolites were only present in small intensities in the metabolite profiles [26]. Due to the nature of the DDA-MS method which preferentially captures the most abundant chemical features, this can explain the low correlation of ecological characteristics with compound classes in our study [26]. Future studies can improve this limitation by utilizing instruments with higher sensitivity which allow to further decrease the fragmentation intensity threshold. This will result in longer measurements and more complex MS2 profiles, but improves the likelihood that more specific secondary metabolites are detected [26].

Bryophytes realize different adaptation strategies to seasonal variations. The responses of bryophytes are likely the result of both their evolutionary history and of adaptations to the local environment. The response of pleurocarpous species was more homogeneous than for acrocarpous species which may be related to their closer phylogenetic relatedness [4,70].

The large chemical richness and contrasting chemodiversity of *Marchantia polymorpha* explained its most basal phylogenetic position and the largest distance of the species in both the chemotaxonomic and the phylogenetic tree [2]. Our results further support that *M. polymorpha* produced many characteristic neolignans (bis-bibenzyls), stilbenes, methoxyphenols, and anthocyanins; and which have only been found in liverworts [8]. Sesquiterpenoids, flavonoids, glycosides, steroids, and lactones were among the most characteristic compound classes in *M. polymorpha*, which explain the strong phylogenetic signal.

Our results show that the different order of the acrocarpous *Polytrichum strictum* in the chemotaxonomic tree is likely due to the many unique chemical features with relatively low peak intensities in the metabolite profiles that caused the DDA-MS to pick fewer features for MS2 fragmentation (especially fewer lipids and lipid-like molecules). Nevertheless, the chemotaxonomic distance of *P. strictum* could also be attributed to a different distribution of compounds in the classes of sesquiterpenoids and phenylpropanoids and the presence of some unique methoxyphenols, anthocyanins, and carbohydrates (including monosaccharides and glycosyl compounds) which were enriched in the winter season. This may be an adaptation of the species to wet habitat conditions.

The results of this study showed that seasonal variations in compound classes were strongly species-specific. The species *M. polymorpha*, *P. undulatum*, and *P. strictum* showed the largest seasonal variations which was likely due to increased bioactivity in the growing seasons and their overall contrasting chemodiversity. Among the best studied compound classes in bryophytes and plants in general are terpenoids, aromatic compounds such as lignans, phenylpropanoids such as flavonoids and anthocyanins, and glycosylic compounds (including flavonoid glycosides) [8,9,71]. Many of the compounds belonging to these classes have been attributed to organismal interactions such as defense against pathogens and herbivores [5,71]. As not all biological antagonists are affected by individual metabolites in the same way, this has led to the chemical richness in secondary metabolites that exist today within plant species [26]. It is thus reasonable to explore whether ecological patterns can be generalized to individual compound classes.

In almost all tested bryophyte species, the number of flavonoids was enriched in spring (except for *M. polymorpha* which produced most in summer). It is widely known that flavonoids constitute pigments which are functioning as light-protection agents in plants [72,73]. As sunlight increases in spring, bryophytes likely produce flavonoids that protect them from excessive light and reactive oxygen species (ROS) which are captured by several types of flavonoids and also anthocyanins. This explains the larger numbers measured in the growing seasons in our study. Flavonoids also regulate enzyme activity and are involved in signal transductions in plant cells [72,74]. In bryophytes, several flavonoids have also been described to play a role in pathogen defense, hence their broad antimicrobial and antifungal bioactivity [8].

The DDA-MS method only detected few methoxyphenols and lactones that were among the most abundant compounds which have otherwise been described to be more common in bryophytes [8]. Methoxyphenols and lactones show bioactivity against pathogens and herbivores [5]. The tested bryophytes produced more compounds of these groups during the growing seasons. Thus, these compounds may be used as biomarkers for organismal interactions of bryophytes. Further investigation is needed.

Similarly, few phenylpropanoid derived cinnamic acids were detected. However, only few cinnamic acids occur naturally. In vascular plants, cinnamic acids are well-known antioxidants [75]. Due to their high redox potential they are also involved in responses against biological antagonists such as pathogens [76]. In bryophytes, however, cinnamic acids may primarily be involved in growth as hydroxylated constituents are part of lignin-like structures (‘proto-lignin’) that have similar functions as in vascular plants and are similarly located in the conducting cell wall tissue of some bryophytes [77,78]. Hydroxylated cinnamic acids as part of proto-lignin are considered to be the building blocks of functional equivalents to phloem in bryophytes called hydroids or leptoids [1,79]. This may also explain the higher peak intensity in the growing seasons in our study. Here, metabolomics can assist to find corresponding genes in bryophytes [78].

**Figure 6:**
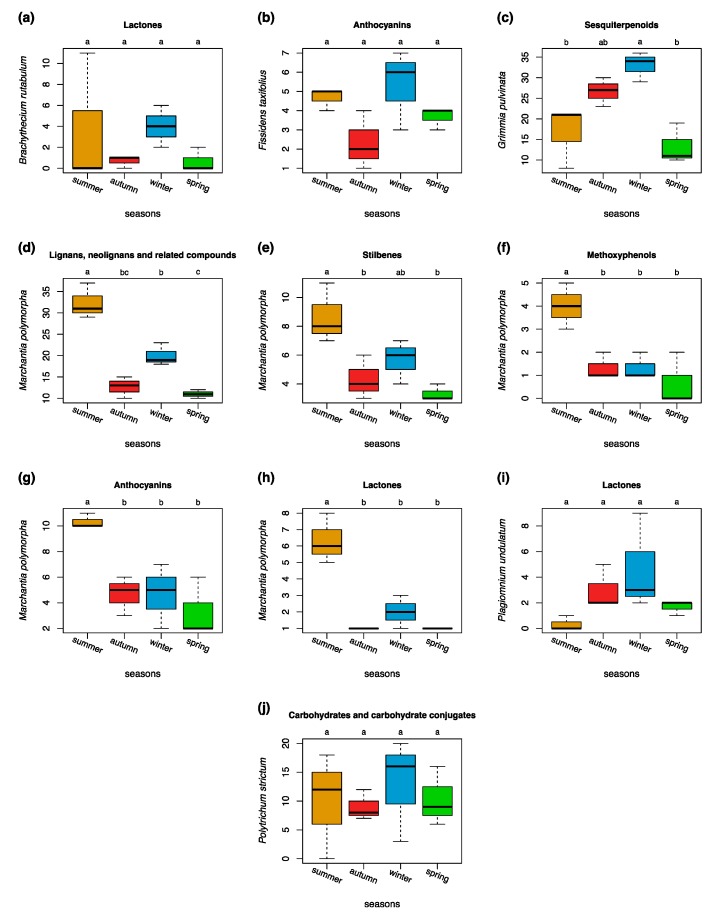
Seasonal variations in selected compound classes of selected species. (**a**) Lactones in *B. rutabulum*, (**b**) anthocyanins in *F. taxifolius*, (**c**) sesquiterpenoids in *G. pulvinata*, (**d**) lignans, neolignans, and related conjugates in *M. polymorpha*, (**e**) stilbenes in *M. polymorpha*, (**f**) methoxyphenols in *M. polymorpha*, (**g**) anthocyanins in *M. polymorpha*, (**h**) lactones in *M. polymorpha*, (**i**) lactones in *P. undulatum*, and (**j**) carbohydrate and carbohydrate conjugates in *P. strictum*. Differences among groups (letters on the top of the plot) are based on the Tukey HSD post hoc test on a one-way ANOVA. Different letters show significant differences (*p* < 0.05). *n* = 27 for each season.

Bryophytes experience cold stress during winter when temperatures are near or below the freezing point. In our study, winder sampling was performed when bryophytes were covered with snow which may have protected them from lowest temperatures [33]. It has been reported that bryophytes are very similar to vascular plants in their response to cold stress. They modify their metabolome (“metabolic reprogramming”) to prevent mechanical damage to the cells [80,81]. Normally, vascular plants upregulate their carbohydrate metabolism in low temperature which results in the production of a variety of different sugars and glycosyl compounds. However, during winter, we detected significantly (*p* < 0.05) more carbohydrates and glycosyl compounds only in the species *P. strictum* and *F. taxifolius* (Figure 6j, Figure S6). Bryophytes may have realized additional adaptation strategies over the production of sugars and other carbohydrates to cold stress and freezing [80]. For *F. taxifolius* and *P. strictum* we detected that they produced more anthocyanins in winter (Figure 6b, Figure S6). Anthocyanins were frequently described to play a protective role against low temperatures in vascular plants [82,83]. The species *B. rutabulum* and *P. undulatum* produced the most lactones in winter (Figure 6a,i). Sesquiterpene lactones can form covalent bonds with proteins and can protect the DNA under physiologically unfavorable conditions [84]. Significantly (*p* < 0.05) more sesquiterpenoids were measured in winter for *R. squarrosum*, *P. undulatum*, and *G. pulvinata* (Figure 6c, Figure S6). Sesquiterpenoids in *M. polymorpha* were also increased in winter when compared to autumn and spring. Thus, bryophytes may survive extended periods of cold stress by the enhanced production of some terpenoids. Only few studies have reported terpenes to play a role during cold stress [85,86,87]. In this regard, bryophytes may be different to vascular plants. Our results could be confounded by other entities belonging to the same compound classes to be produced as part of desiccation tolerance [22,23,88,89]. Nevertheless, this finding warrants further investigation in bryophytes.

For compound annotation and classification, spectral libraries such as the WEIZMASS spectral library [90], GNPS [91], or MassBank [41] are necessary that contain a magnitude of MS2 fragment spectra (for an overview and comparison of spectral libraries, see the review [92]). However, many of these libraries are biased towards humans or model-species such as *Arabidopsis thaliana*. On one hand, some compound classes where there is a lot of research can be overrepresented in libraries. On the other hand, even specialized libraries often do not contain information on non-model species typically used in ecometabolomics [13,93]. Furthermore, not all spectral libraries are also openly available. These challenges often impede both the classification and the identification in ecometabolomics due to the lack of constitutive reference compound spectra. This is likely an explanation why our classifier could not assign chemical entities into the relatively common classes of diterpenoids, benzene and substituted derivatives. To build classifiers, we used fragment spectra from the openly available database MassBank [41]. However, as MassBank did not contain spectra of (neo)lignans, bis-bibenzyls, and only few sesquiterpenoids, we complemented the library with manually curated spectra from known compounds of *M. polymorpha* (To this end, a pull request has been created in the MassBank GitHub repository: https://github.com/MassBank/MassBank-data/pull/78) [41,93]. To promote ecometabolomics, we want to raise the awareness of spectral data in open libraries and want to encourage fellow researchers to contribute MS2 spectra of bryophytes or other non-model organisms.

To assess the chemical diversity and its ecological relevance it is necessary to delve into the “dark matter” to explore the magnitude of completely novel and unknown natural products and to relate them to ecological functioning [26,34]. Our study presents a novel methodology to automatically analyze the most abundant secondary metabolites based on *in silico* compound classification. We present a methodological framework to automate compound classification which facilitates complex biochemical analyses and enables interpretation based on ecological parameters and classification to be used in hypotheses generation. In order to be reused by future ecometabolomics studies we made the representative dataset available as MTBLS709 in the MetaboLights repository [56] and also the computational code which can be used in other studies. We inspire future ecometabolomics studies to adopt and improve our framework to allow more detailed insights into the ecological roles of biochemical constituents of bryophytes.

## Figures and Tables

**Table 1:** List of classified compound classes and the corresponding area under precision–recall curve (AUC-PR) and the true positive rate for a fixed false negative rate of 5% (TPR-FNR) determined by our classifier in all of the bryophyte samples. The higher the AUC-PR value the better was the performance of the classifier. Generally, values above 0.5 are considered to be a good performance metric with imbalanced data [53]. The higher the TPR-FNR the larger was for the probability that the classifier assigned chemical entities correctly to a compound class. A value of 0 means for both metrics that the classifier could not assign enough spectra to a particular compound class.

Compound Class	ChemOnt ID	AUC-PR	TPR-FNR
Lipids and lipid-like molecules	0000012	0.347	0.257
Fatty acids and conjugates	0000262	0.170	0.357
Steroids and steroid derivatives	0000258	0.511	0.639
Prenol lipids	0000259	0.139	0.375
Monoterpenoids	0001549	0	0
Sesquiterpenoids	0001550	0.178	0.667
Diterpenoids	0001551	0	0
Triterpenoids	0001553	0	0
Benzene and substituted derivatives	0000178	0	0
Benzoic acids	0002565	0	0
Phthalic acid derivates	0001105	0	0
Lignans, neolignans, and related compounds	0001392	0.722	0.750
Lactones	0000050	0.133	0.224
Phenylpropanoids and polyketides	0000261	0.450	0.277
Cinnamic acids	0002504	0.024	0.333
Coumarins and derivatives	0000145	0	0
Stilbenes	0000253	0.028	0.250
Methoxyphenols	0000190	0.085	0.321
Flavonoids	0000334	0.587	0.656
Biflavonoids and polyflavonoids	0001586	0	0
Flavones	0001615	0.465	0.636
Flavans	0000337	0.094	0.429
Flavonoid glycosides	0001111	0.816	0.897
Anthocyanins	0001361	0.297	1.000
Carbohydrates and carbohydrate conjugates	0001542	0.594	0.567
Monosaccharides	0001540	0.398	0.490
Disaccharides	0001542	0.465	0.643
Oligosaccharides	0000198	0	0
Polysaccharides	0001539	0	0
Glycosyl compounds	0002105	0.619	0.598
Alkaloids and derivatives	0000279	0.192	0.286
Organic acids and derivatives	0000264	0	0
Amino acids	0004176	0.276	0.288
Peptides	0000348	0.092	0.200
Nucleotides	0000289	0.311	0.583

## Data Accessibility

Raw Metabolite profiles, metabolite feature matrices, MS2 fragment spectra, classifiers and meta-data: MetaboLights MTBLS709 (https://www.ebi.ac.uk/metabolights/MTBLS709). Computational code to reproduce the results of this study. Version 1.1. Zenodo doi:10.5281/zenodo.3478217.

## Supplementary Materials

The following are available online at https://www.mdpi.com/2218-1989/9/10/222/s1, Figure S1: Comparison of three diversity indices of the bryophyte species, Figure S2: Comparison of three indices that explain the biochemical diversity of the bryophytes with regard to the four seasons, Figure S3: Variation partitioning of the study factors species and seasons of the (a) MS1 feature matrix, (b) richness of chemical entities in the classified compound classes, Figure S4: Richness of chemical entities for the different bryophyte species within the chemical classes of (a) Lipids and lipid-like molecules, (b) Fatty acids and conjugates, (c) Steroids and steroid derivatives, (d) Prenol lipids, (e) Sesquiterpenoids, (f) Lignans, neolignans and related compounds, (g) Lactones, (h) Phenylpropanoids and polyketides, (i) Cinnamic acids, (j) Stilbenes, (k) Methoxyphenols, (l) Flavonoids, (m) Flavones, (n) Flavans, (o) Flavonoid glycosides, (p) Anthocyanins, (q) Carbohydrates and carbohydrate conjugates, (r) Monosaccharides, (s) Disaccharides, (t) Glycosyl compounds, (u) Alkaloids and derivatives, (v) Amino acids, (w) Peptides, and (x) Nucleosides, nucleotides, and analogues, Figure S5: Seasonal variations in the chemical richness of the tested bryophyte species within the chemical classes of (a) Lipids and lipid-like molecules, (b) Fatty acids and conjugates, (c) Steroids and steroid derivatives, (d) Prenol lipids, (e) Sesquiterpenoids, (f) Lignans, neolignans and related compounds, (g) Lactones, (h) Phenylpropanoids and polyketides, (i) Cinnamic acids, (j) Stilbenes, (k) Methoxyphenols, (l) Flavonoids, (m) Flavones, (n) Flavans, (o) Flavonoid glycosides, (p) Anthocyanins, (q) Carbohydrates and carbohydrate conjugates, (r) Monosaccharides, (s) Disaccharides, (t) Glycosyl compounds, (u) Alkaloids and derivatives, (v) Amino acids, (w) Peptides, and (x) Nucleosides, nucleotides, and analogues, Figure S6: Seasonal variations in compound classes of the tested bryophyte species, Figure S7: Quality control as employed by the mzQuality workflow, Figure S8: Total ion chromatograms for the nine tested species grouped by seasons, Table S1: Explained seasonal variation in the compound classes by the different bryophyte species, Table S2: Influence of ecological characteristics on the matrix of compound classes.
