# Supplementary material for: Chemical Diversity and Classification of Secondary Metabolites in Nine Bryophyte Species
Source: Metabolites. 2019 Oct 11;9(10):222. doi: 10.3390/metabo9100222 (PMC6835487; doi:10.3390/metabo9100222)
Supplement: Supplementary file 1 [file metabolites-09-00222-s001.zip › fig_3.pdf]

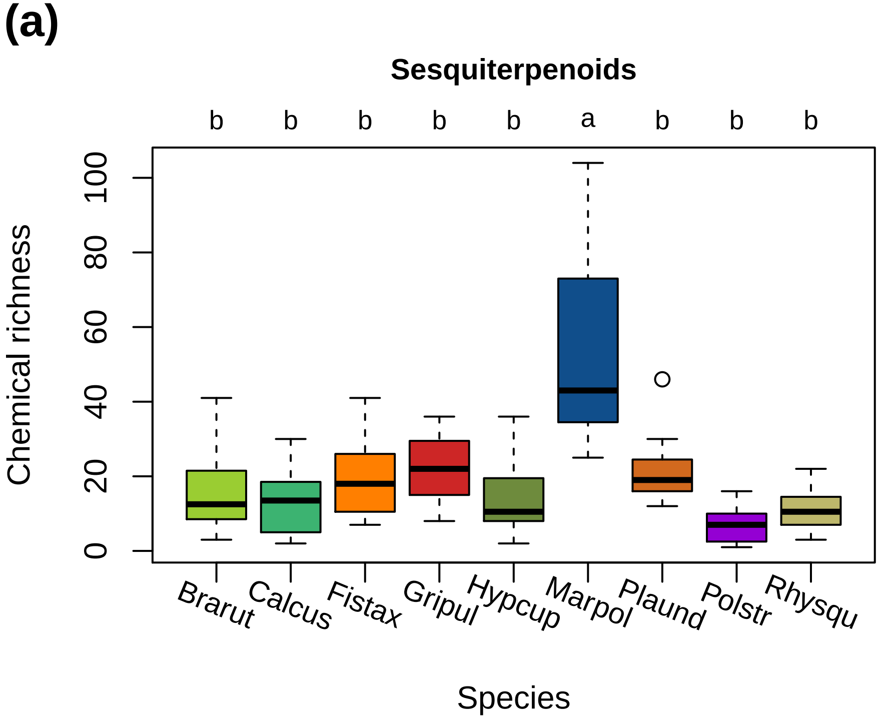
(a)
Sesquiterpenoids
b b b b b a b b b
Chemical richness
0
20
40
60
80
100
Brarut
Calcus
Fistax
Gripul
Hypcup
Marpol
Plaund
Polstr
Rhysqu
Species

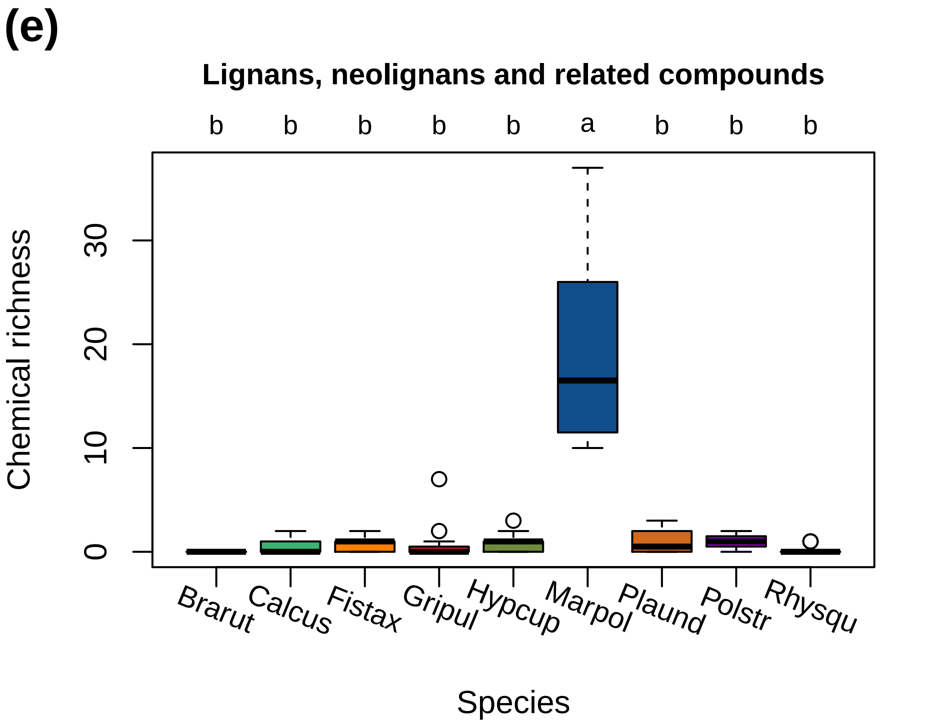
(e)
Lignans, neolignans and related compounds
b b b b b a b b b
Chemical richness
0
10
20
30
Brarut
Calcus
Fistax
Gripul
Hypcup
Marpol
Plaund
Polstr
Rhysqu
Species

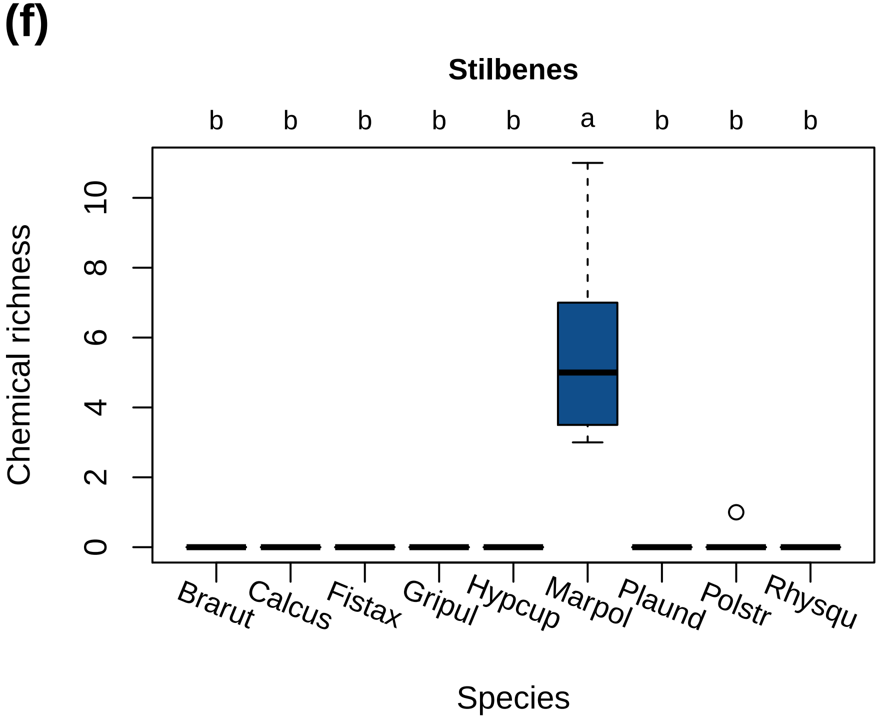
(f)
Stilbenes
b
b
b
b
b
a
b
b
b
Chemical richness
0
2
4
6
8
10
Brarut
Calcus
Fistax
Gripul
Hypcup
Marpol
Plaund
Polstr
Rhysqu
Species

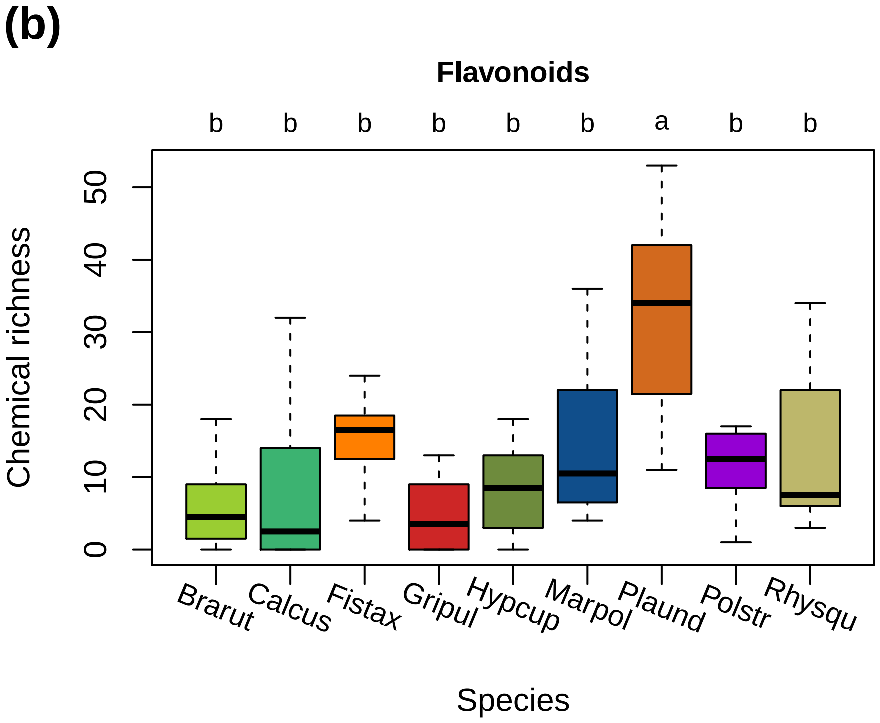

(b)
Flavonoids
b b b b b b a b b
Chemical richness
0 10 20 30 40 50
Brarut Calcus Fistax Gripul Hypcup Marpol Plaund Polstr Rhysqu
Species

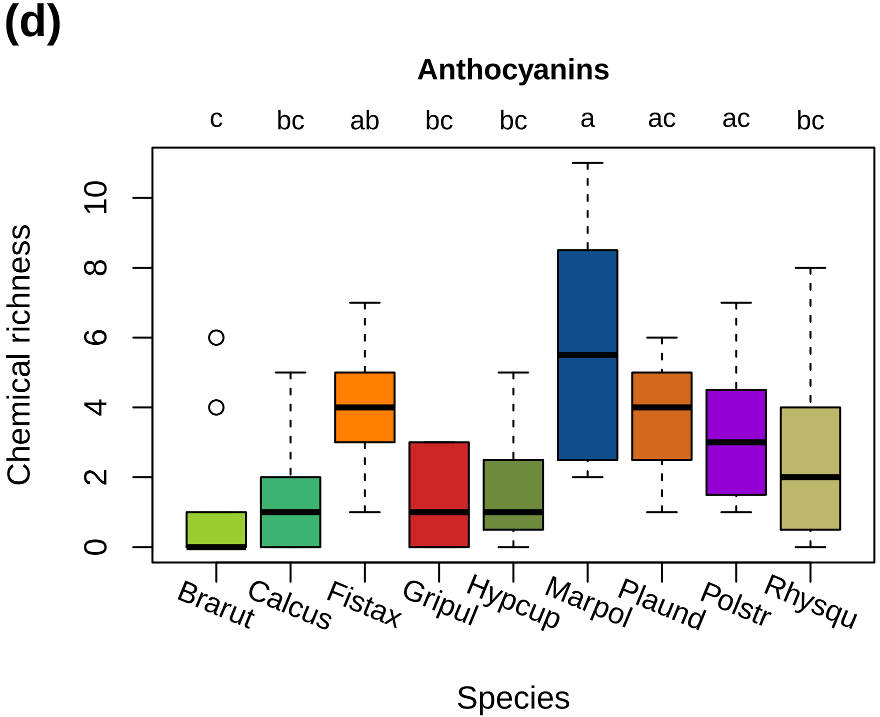
(d)
Anthocyanins
c
bc
ab
bc
bc
a
ac
ac
bc
Chemical richness
0
2
4
6
8
10
Brarut
Calcus
Fistax
Gripul
Hypcup
Marpol
Plaund
Polstr
Rhysqu
Species

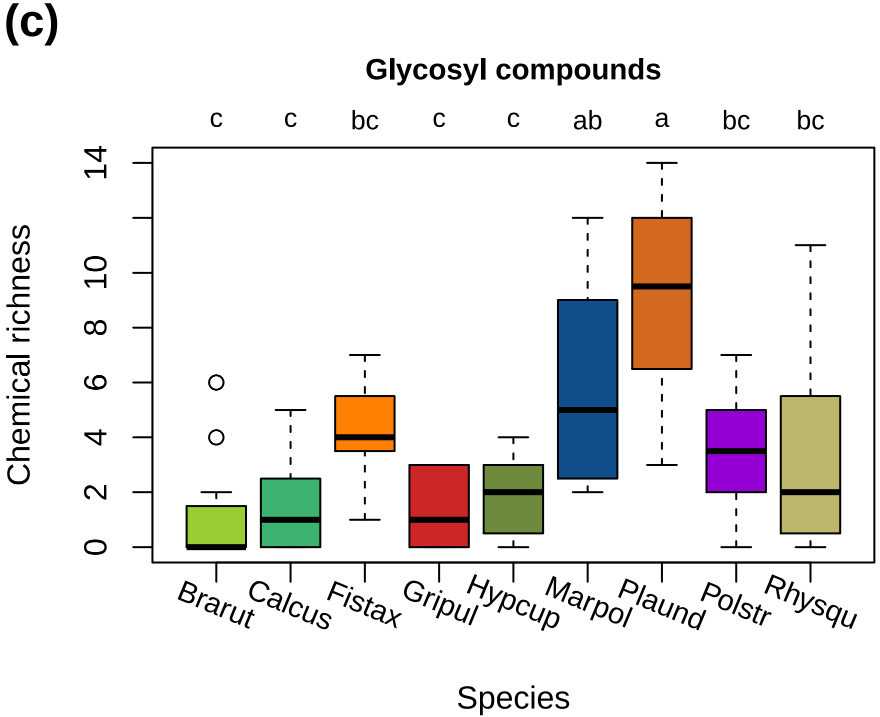
(c)
Glycosyl compounds
c
c
bc
c
c
ab
a
bc
bc
Chemical richness
0
2
4
6
8
10
14
Brarut
Calcus
Fistax
Gripul
Hypcup
Marpol
Plaund
Polstr
Rhysqu
Species
