# Supplementary material for: Chemical Diversity and Classification of Secondary Metabolites in Nine Bryophyte Species
Source: Metabolites. 2019 Oct 11;9(10):222. doi: 10.3390/metabo9100222 (PMC6835487; doi:10.3390/metabo9100222)
Supplement: Supplementary file 1 [file metabolites-09-00222-s001.zip › fig_5.pdf]

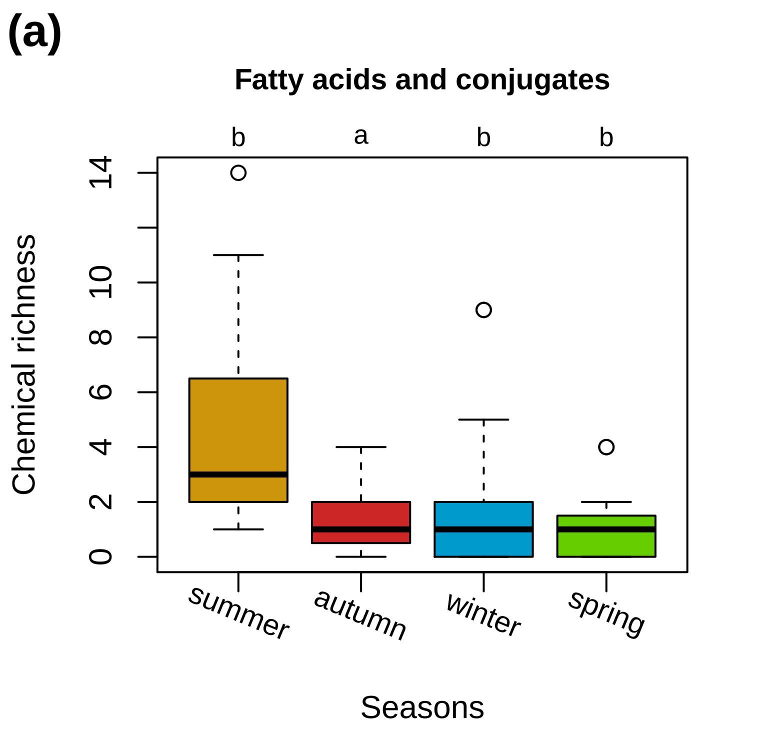
(a)
Fatty acids and conjugates
b
a
b
b
14
10
8
6
4
2
0
Chemical richness
summer
autumn
winter
spring
Seasons

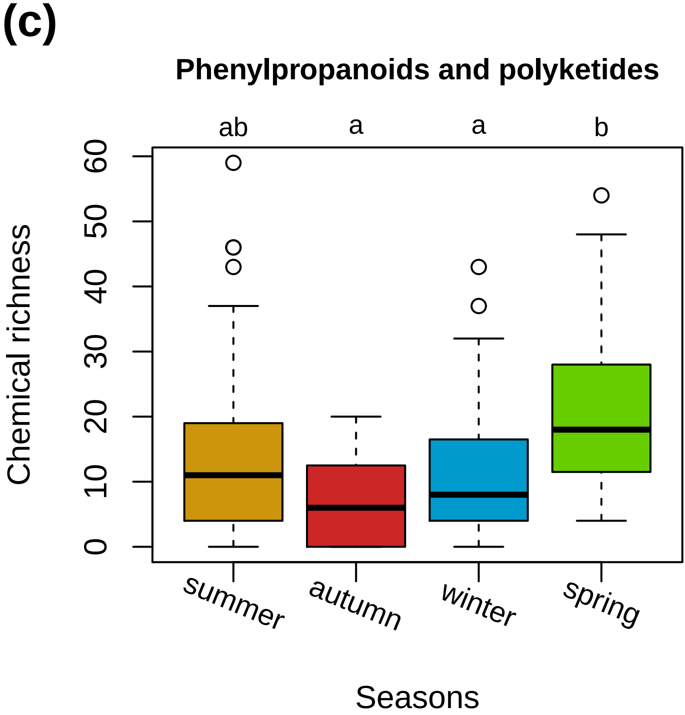

(c)
Phenylpropanoids and polyketides
ab
a
a
b
Chemical richness
0
10
20
30
40
50
60
summer
autumn
winter
spring
Seasons

(d)

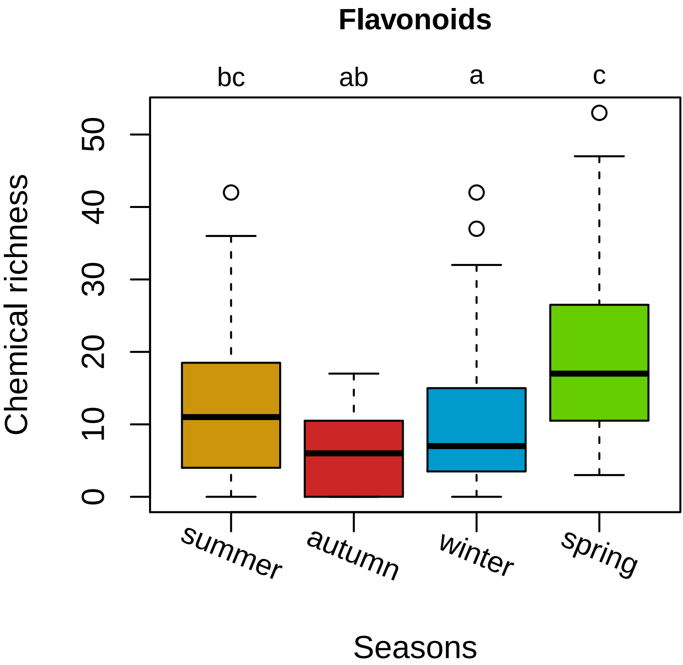

Flavonoids
bc
ab
a
c
Chemical richness
0
10
20
30
40
50
summer
autumn
winter
spring
Seasons

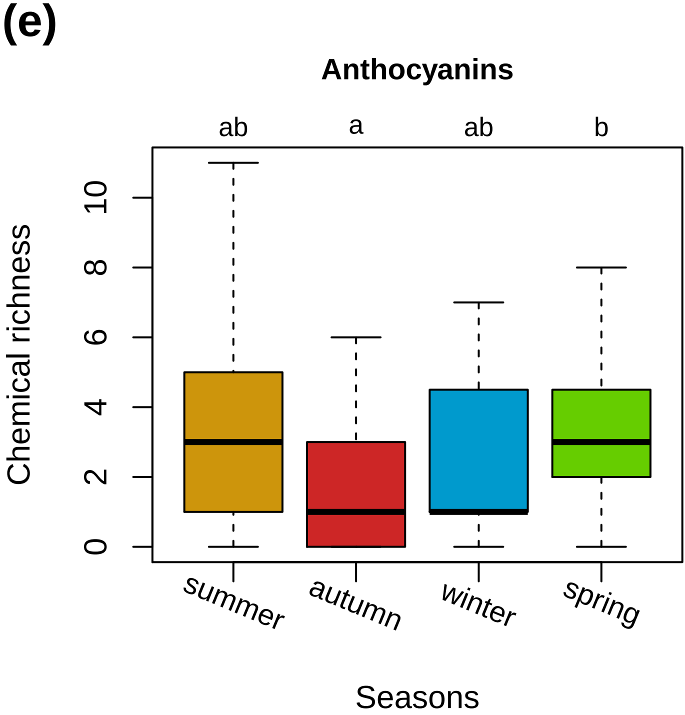
(e)
Anthocyanins
ab
a
ab
b
Chemical richness
0
2
4
6
8
10
summer
autumn
winter
spring
Seasons

(b)

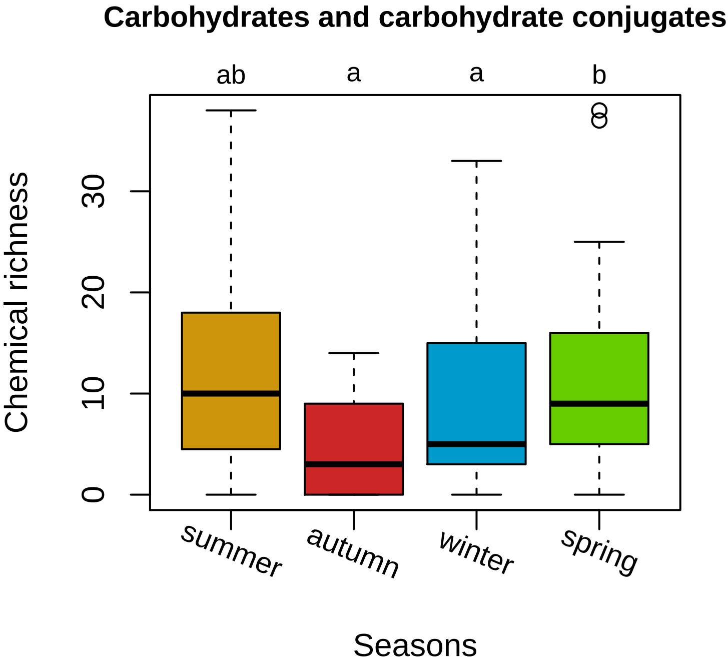

Carbohydrates and carbohydrate conjugates
ab
a
a
b
Chemical richness
0
10
20
30
summer
autumn
winter
spring
Seasons
