# Supplementary material for: Chemical Diversity and Classification of Secondary Metabolites in Nine Bryophyte Species
Source: Metabolites. 2019 Oct 11;9(10):222. doi: 10.3390/metabo9100222 (PMC6835487; doi:10.3390/metabo9100222)
Supplement: Supplementary file 1 [file metabolites-09-00222-s001.zip › fig_6.pdf]

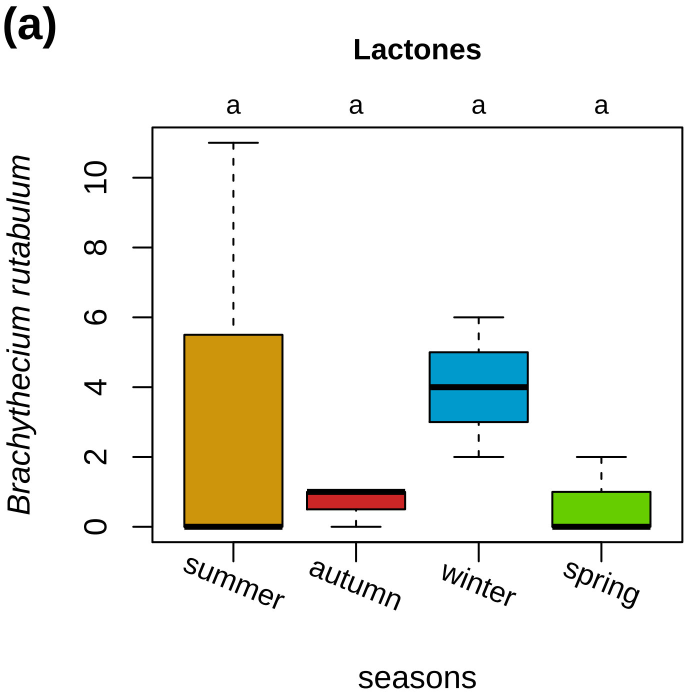

(a)
Lactones
a
a
a
a
Brachythecium rutabulum
0
2
4
6
8
10
summer
autumn
winter
spring
seasons

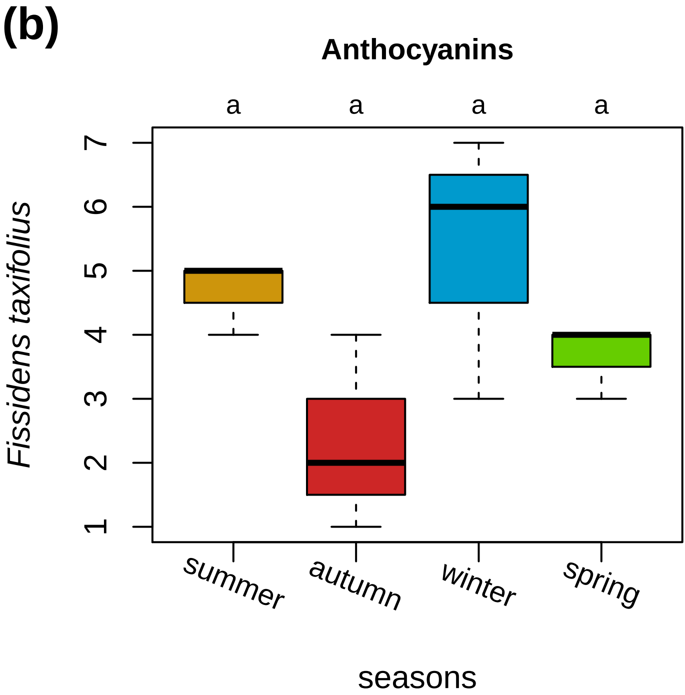

(b)
Anthocyanins
a
a
a
a
Fissidens taxifolius
1
2
3
4
5
6
7
summer
autumn
winter
spring
seasons

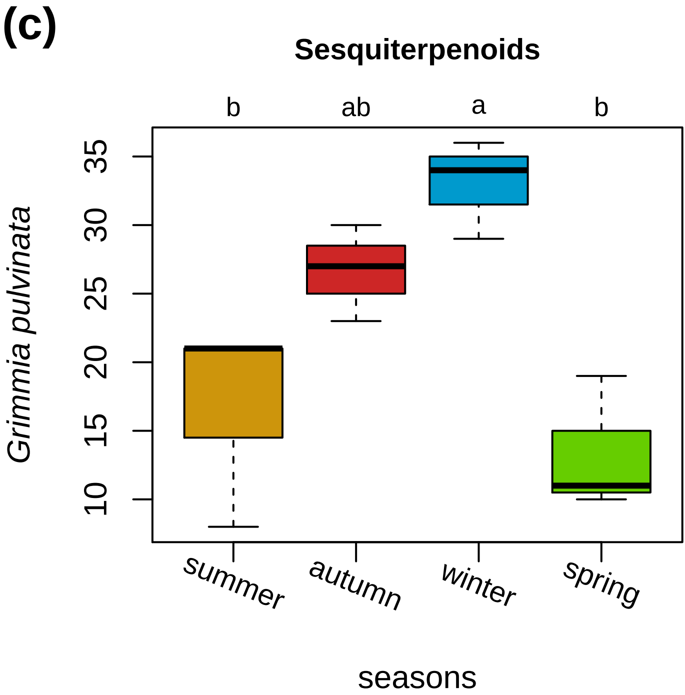
(c)
Sesquiterpenoids
b
ab
a
b
35
30
25
20
15
10
Grimmia pulvinata
summer
autumn
winter
spring
seasons

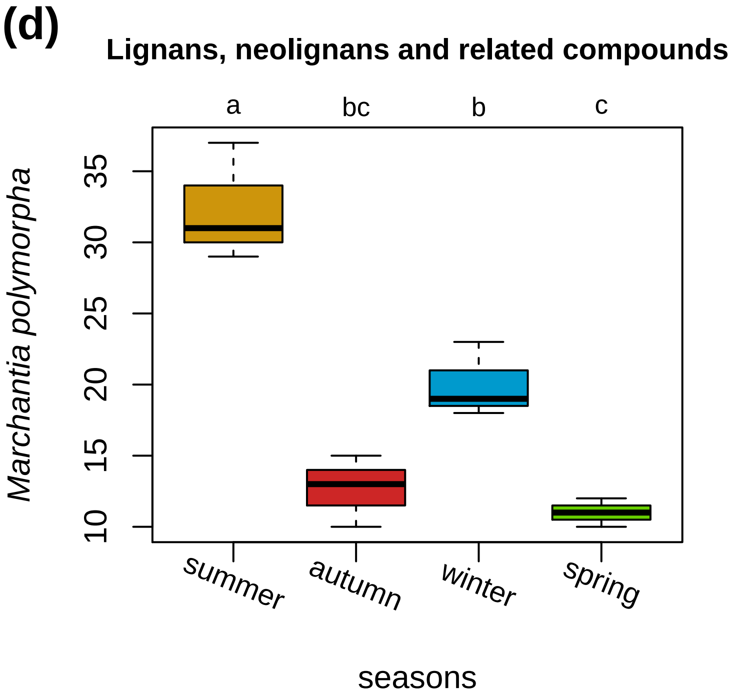
(d)
Lignans, neolignans and related compounds
a
bc
b
c
Marchantia polymorpha
10
15
20
25
30
35
summer
autumn
winter
spring
seasons

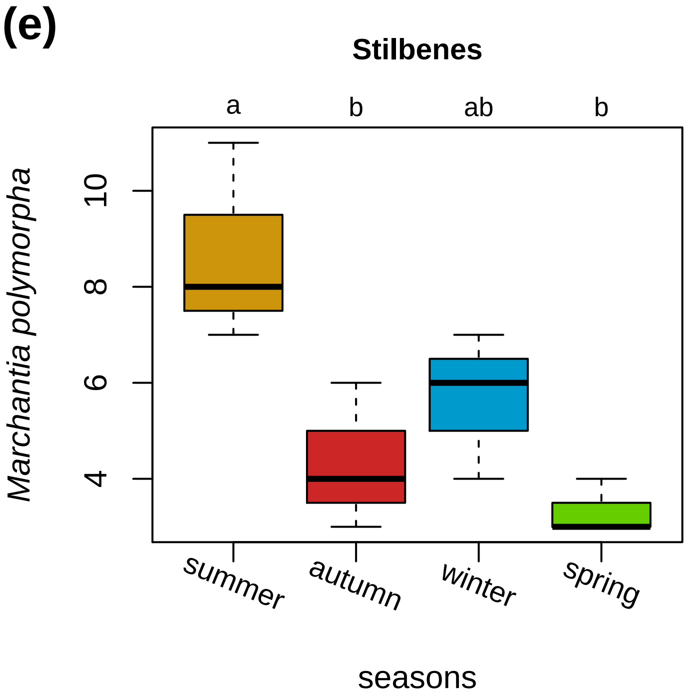

(e)
Stilbenes
a
b
ab
b
Marchantia polymorpha
10
8
6
4
summer
autumn
winter
spring
seasons

(f)

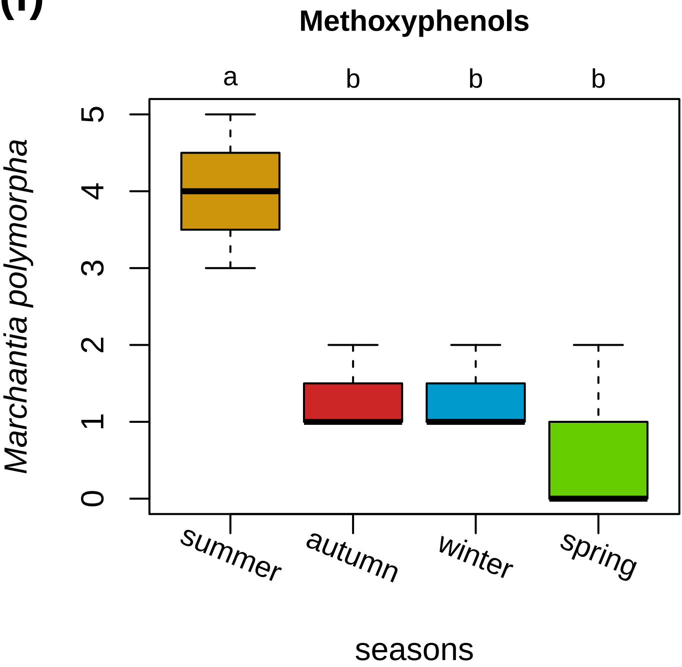

Methoxyphenols
a
b
b
b
5
4
3
2
1
0
Marchantia polymorpha
summer
autumn
winter
spring
seasons

(g)

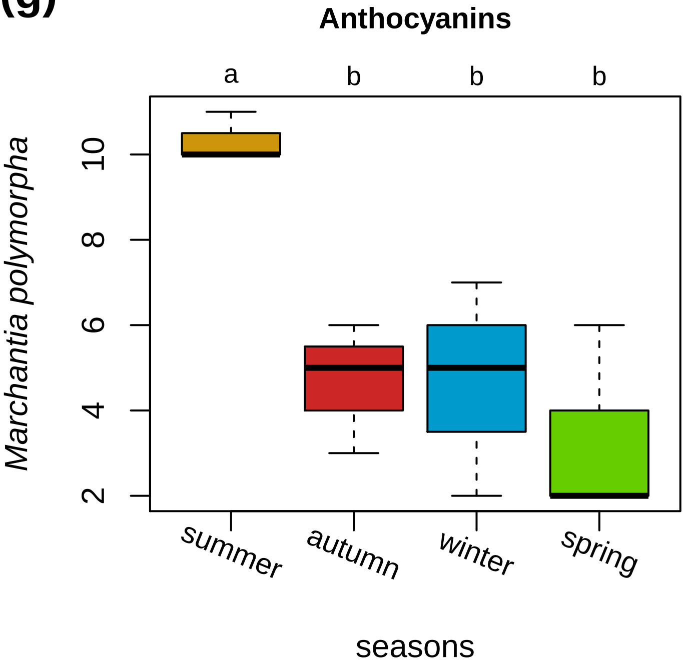
Anthocyanins
a
b
b
b
10
8
6
4
2
Marchantia polymorpha
summer
autumn
winter
spring
seasons

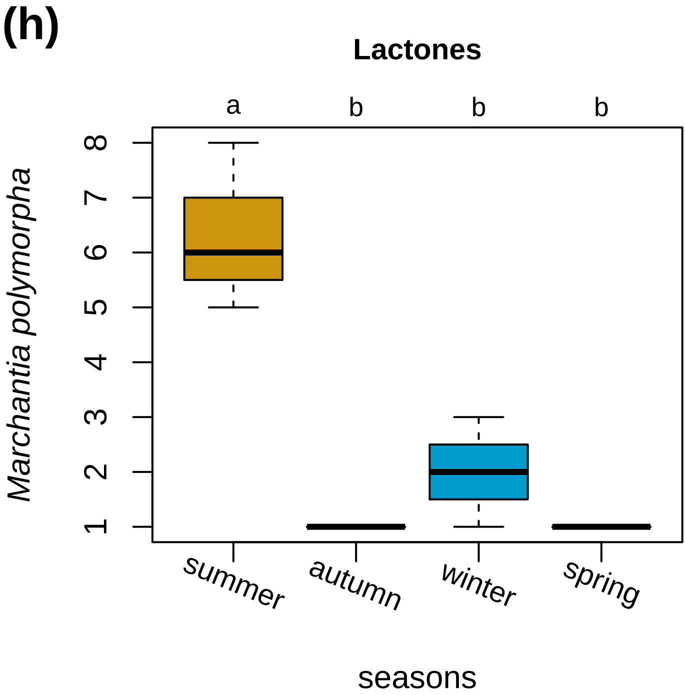

(h)
Lactones
a
b
b
b
Marchantia polymorpha
1
2
3
4
5
6
7
8
summer
autumn
winter
spring
seasons

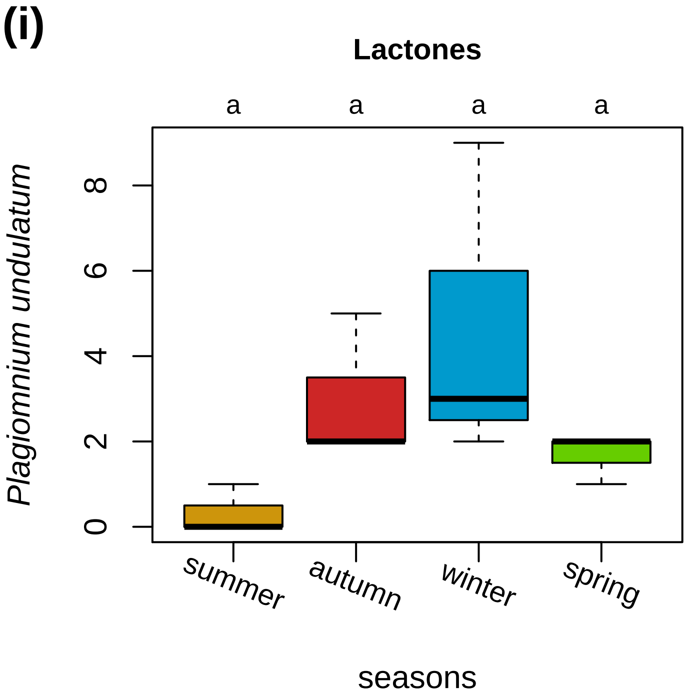
(i)
Lactones
a
a
a
a
Plagiomnium undulatum
0
2
4
6
8
summer
autumn
winter
spring
seasons

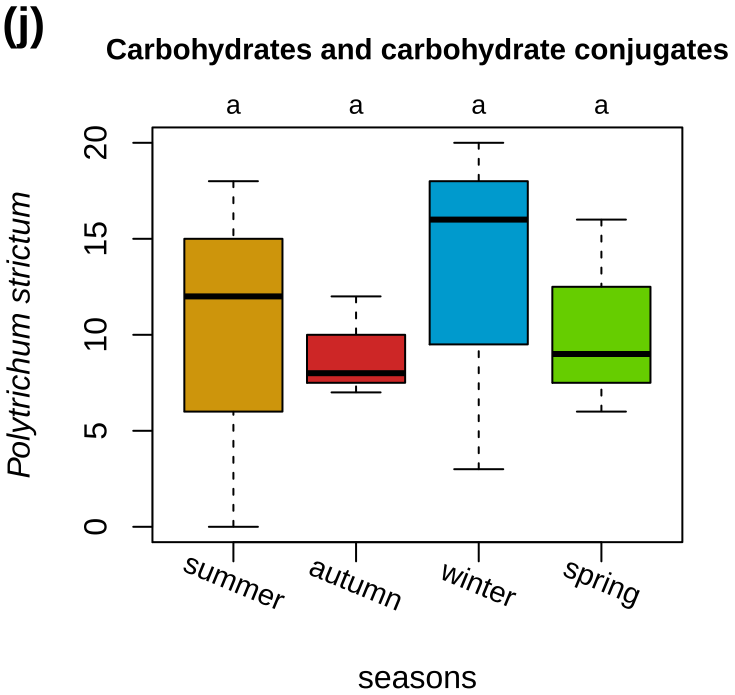

(j)
Carbohydrates and carbohydrate conjugates
a
a
a
a
Polytrichum strictum
0
5
10
15
20
summer
autumn
winter
spring
seasons
