# Supplementary material for: Chemical Diversity and Classification of Secondary Metabolites in Nine Bryophyte Species
Source: Metabolites. 2019 Oct 11;9(10):222. doi: 10.3390/metabo9100222 (PMC6835487; doi:10.3390/metabo9100222)
Supplement: Supplementary file 1 [file metabolites-09-00222-s001.zip › supporting-information.docx]

**Supplemental Material**

***Diversity of secondary metabolites at MS1 level***

To assess the diversity, the LC/MS raw data was processed at MS1 level which resulted in a matrix with 7,044 features across a total of 108 samples. Variation partitioning showed that that the study factor species accounted for a total of 30% of variation in the MS1 feature matrix and the factor seasons explained 9% of variation (Fig. S3a).

In the metabolite profiles of *Marchantia polymorpha* there were significantly (p<0.05) more biochemical entities (features) present with a higher intensities than in the other species (Fig. S1a,d). This also resulted in a significantly (p<0.05) larger diversity of compounds which were more unevenly distributed than in the profiles of other species mainly due to differently composed profiles in the summer season (Fig 1b,c). In general, metabolite profiles of pleurocarpous mosses had fewer features which resulted in a relatively low Shannon diversity index for this group of mosses (Fig. S1b). By contrast, acrocarpous mosses had more features, with *Plagiomnium undulatum* having the most features with higher intensities in this group and *Polytrichum strictum* the least diversity and least peak intensities in this group but not in the unique features (Fig. S1a-d).

**Figure S1**: Comparison of three diversity indices of the bryophyte species. Differences among groups (letters on the top of the plot) are based on the Tukey HSD post hoc test on a one-way ANOVA. Different letters show significant differences (p<0.05). n = 12 for each species. Red colors represent acrocarpous mosses. Green colors represent pleurocarpous mosses. A blue color represents liverworts. (a) Number of unique chemical entities (features) that were only present in one of the nine species. (b) Shannon diversity index *H’* to assess the features richness and their distribution among the species. (c) Pielou’s diversity index *J* to estimate the evenness of the distribution of features among the species. (d) Total intensities (sum of total ion current, TIC) of the species samples.

In the autumn season there were significantly (p<0.05) fewer chemical entities (features) present with lower peak intensities than in the other seasons (Fig. S2b,d). This resulted in a significantly (p<0.05) lower Shannon diversity index and more evenly distributed profiles in the autumn season (Fig. S2b,c). The highest chemical diversity in both the number of features and intensity was observed in the spring and summer growing seasons (Fig. S2b-d).

**Figure S2**: Comparison of three indices that explain the biochemical diversity of the bryophytes with regard to the four seasons. Differences among groups (letters on the top of the plot) are based on the Tukey HSD post hoc test on a one-way ANOVA. Different letters show significant differences (p<0.05). n = 27 for each season. (a) Number of unique features that were only detected in one of the seasons. (b) Shannon diversity index (H’) for chemical entities of the metabolite profiles for the seasons. (c) Pielou’s diversity index *J* to estimate the evenness of the features distribution among the seasons. (d) Total intensities (sum of total ion current, TIC) of the season samples.

***Classification of secondary metabolites into compound classes***

**Figure S3**: Variation partitioning of the study factors species and seasons of the (a) MS1 feature matrix, (b) richness of chemical entities in the classified compound classes.

**Table S1**: Explained seasonal variation in the compound classes by the different bryophyte species.

| **Species** | **r^2^** | **p-value** |
| --- | --- | --- |
| *Brachythecium rutabulum* | 0,369 | 0,005 |
| *Calliergonella cuspidata* | 0,355 | 0,006 |
| *Fissidens taxifolius* | 0,188 | 0,478 |
| *Grimmia pulvinata* | 0,245 | 0,141 |
| *Hypnum cupressiforme* | 0,355 | 0,004 |
| *Marchantia polymorpha* | 0,808 | 0,001 |
| *Plagiomnium undulatum* | 0,620 | 0,001 |
| *Polytrichum strictum* | 0,490 | 0,001 |
| *Rhytidiadelphus squarrosus* | 0,240 | 0,148 |

***Species-specific variations in the compound classes***

**Figure S4:** Richness of chemical entities for the different bryophyte species within the chemical classes of (a) Lipids and lipid-like molecules, (b) Fatty acids and conjugates, (c) Steroids and steroid derivatives, (d) Prenol lipids, (e) Sesquiterpenoids, (f) Lignans, neolignans and related compounds, (g) Lactones, (h) Phenylpropanoids and polyketides, (i) Cinnamic acids, (j) Stilbenes, (k) Methoxyphenols, (l) Flavonoids, (m) Flavones, (n) Flavans, (o) Flavonoid glycosides, (p) Anthocyanins, (q) Carbohydrates and carbohydrate conjugates, (r) Monosaccharides, (s) Disaccharides, (t) Glycosyl compounds, (u) Alkaloids and derivatives, (v) Amino acids, (w) Peptides, and (x) Nucleosides, nucleotides, and analogues. Differences among groups (letters on the top of the plot) are based on the Tukey HSD post hoc test on a one-way ANOVA. Different letters show significant differences (p<0.05). n = 12 for each species. Red colors represent acrocarpous mosses. Green colors represent pleurocarpous mosses. A blue color represents liverworts.

***Seasonal variations in the compound classes***

**Figure S5:** Seasonal variations in the chemical richness of the tested bryophyte species within the chemical classes of (a) Lipids and lipid-like molecules, (b) Fatty acids and conjugates, (c) Steroids and steroid derivatives, (d) Prenol lipids, (e) Sesquiterpenoids, (f) Lignans, neolignans and related compounds, (g) Lactones, (h) Phenylpropanoids and polyketides, (i) Cinnamic acids, (j) Stilbenes, (k) Methoxyphenols, (l) Flavonoids, (m) Flavones, (n) Flavans, (o) Flavonoid glycosides, (p) Anthocyanins, (q) Carbohydrates and carbohydrate conjugates, (r) Monosaccharides, (s) Disaccharides, (t) Glycosyl compounds, (u) Alkaloids and derivatives, (v) Amino acids, (w) Peptides, and (x) Nucleosides, nucleotides, and analogues. Differences among groups (letters on the top of the plot) are based on the Tukey HSD post hoc test on a one-way ANOVA. Different letters show significant differences (p<0.05). n = 27 for each season.

***Seasonal variations in compound classes in different bryophyte species***

*B. rutabulum* and *R. squarrosus* showed enhanced production of lipids, steroids and sesquiterpenoids in winter. *B. rutabulum* produced more fatty acids in summer and autumn. Profiles of *C. cuspidata* had significantly (p<0.05) fewer lipids, steroids, sesquiterpenoids, phenylpropanoids, carbohydrates and anthocyanins in autumn and most in spring. *H. cupressiforme* produced more lipids, fatty acids, steroids, sesquiterpenoids, flavonoids, carbohydrates, anthocyanins in spring and summer than in the other seasons. The species *R. squarrosum* produced most fatty acids in summer and most flavonoids, carbohydrates (and sugars) anthocyanins and methoxyphenols in spring (Fig. S6a,b,e,i).

The acrocarpous species *F. taxifolius* and *P. strictum* produced many flavonoids, anthocyanins, carbohydrates (and sugars) in winter, whereas for *P. strictum* this was also true for the summer season. The species *G. pulvinata* and *P. undulatum* had enriched lipids, steroids and sesquiterpenoids production in winter. For *F. taxifolius* and *P. strictum* this relationship was reversed in the growing seasons spring and summer, where they produced most lipids, steroids and sesquiterpenoids. *G. pulvinata* and *P. undulatum* had enriched flavonoid and phenylpropanoid production in spring. *P. undulatum* produced many anthocyanins and glycosyl compounds in the growing seasons spring and summer (Fig. S6c,d,g,h).

The liverwort *M. polymorpha* produced most lipids, fatty acids, steroids, sesquiterpenoids, lignans, lactones, cinnamic acids, stilbenes, methoxyphenols, flavonoids, anthocyanins and carbohydrates in summer; and the fewest lignans, lipids, stilbenes, steroids and phenylpropanoids in autumn and spring (Fig. S6f).

**Figure S6**: Seasonal variations in compound classes of the tested bryophyte species. Differences among groups (letters on the top of the plot) are based on the Tukey HSD post hoc test on a one-way ANOVA. Different letters show significant differences (p<0.05). n = 27 for each season. (a) *Brachythecium rutabulum*. (b) *Calliergonella cuspidata*. (c) *Fissidens taxifolius*. (d) *Grimmia pulvinata*. (e) *Hypnum cupressiforme*. (f) *Marchantia polymorpha*. (g) *Plagiomnium undulatum*. (h) *Polytrichum strictum*. (i) *Rhytidiadelphus squarrosus*.

***Influence of ecological characteristics on the compound classes***

Fitting the ecological characteristics on the matrix of compound classes revealed that a wet moisture status during sampling (on-site moisture level: wet) had an effect on the composition of compound classes (Table S2).

**Table S2**: Influence of ecological characteristics on the matrix of compound classes. r^2^ and p-values were calculated by fitting the ecological characteristics post-hoc on the dbRDA ordination of the compound classes using the function envfit in the package vegan. The table shows the ecological characteristics and corresponding goodness of fit statistics r^2^ and empirical p-values. The study factors species and seasons were included in the model. p-values less than 0.05 are marked in bold font.

| **Trait** | **r^2^** | **p-value** |
| --- | --- | --- |
| Phylogenetic type: Acrocarpous | 0,0144 | 0,4575 |
| Phylogenetic type: Pleurocarpous | 0,0000 | 1,0000 |
| Phylogenetic type: Liverwort | 0,0185 | 0,3636 |
| Growth form: Turf | 0,0139 | 0,4725 |
| Growth form: Mat | 0,0091 | 0,6414 |
| Growth form: Cushion | 0,0084 | 0,6344 |
| Growth form: Dendroid | 0,0185 | 0,3636 |
| Growth form: Thalloid | 0,0024 | 0,8891 |
| Habitat type: Woods, Shrubs | 0,0139 | 0,4725 |
| Habitat type: Exposed Rocks | 0,0295 | 0,2088 |
| Habitat type: Meadows, Herbaceous | 0,0185 | 0,3636 |
| Habitat type: Ruderal, Banks | 0,0054 | 0,7463 |
| Substrate: Soil | 0,0029 | 0,8531 |
| Substrate: Turf, Soil | 0,0139 | 0,4725 |
| Substrate: Dead wood, Bark | 0,0025 | 0,8911 |
| Substrate: Firm rocks | 0,0018 | 0,9071 |
| Substrate: Soil, Turf | 0,0185 | 0,3636 |
| Substrate: Soil, Firm rocks | 0,0277 | 0,2188 |
| Substrate: Soil, Loose rocks | 0,0061 | 0,7373 |
| Life strategy: Colonist | 0,0000 | 1,0000 |
| Life strategy: Perennial stayer competitive | 0,0287 | 0,2438 |
| Life strategy: Perennial stayer stress tolerant | 0,0091 | 0,6414 |
| Life strategy: Pioneer | 0,0306 | 0,1868 |
| Life strategy: Long-lived shuttle | 0,0029 | 0,8531 |
| Gametangia distribution: Autoicous | 0,0292 | 0,2208 |
| Gametangia distribution: Dioicous | 0,0000 | 1,0000 |
| Gametangia distribution: Synoicous | 0,0260 | 0,2318 |
| Mean spore size | 0,0266 | 0,2458 |
| Sexual reproduction frequency: Occasional | 0,0000 | 1,0000 |
| Sexual reproduction frequency: Common | 0,0125 | 0,5065 |
| Sexual reproduction frequency: Very common | 0,0005 | 0,9730 |
| Sexual reproduction frequency: Rare | 0,0085 | 0,6434 |
| Ellenberg light index | 0,0085 | 0,6274 |
| Ellenberg temperature index | 0,0335 | 0,1838 |
| Ellenberg continentality index | 0,0116 | 0,5514 |
| Ellenberg moisture index | 0,0272 | 0,2378 |
| Ellenberg reaction index | 0,0070 | 0,6993 |
| Ellenberg nitrogen index | 0,0016 | 0,9221 |
| Ellenberg life form index: H | 0,0131 | 0,5025 |
| Ellenberg life form index: C, E | 0,0029 | 0,8531 |
| Ellenberg life form index: C | 0,0018 | 0,9071 |
| Ellenberg life form index: H, C | 0,0024 | 0,8891 |
| Ellenberg life form index: C,(E) | 0,0091 | 0,6414 |
| Ellenberg life form index: T | 0,0185 | 0,3636 |
| Onsite substrate: Soil | 0,0343 | 0,1359 |
| Onsite substrate: Rock | 0,0016 | 0,9211 |
| Onsite substrate: Rock and lean soil | 0,0136 | 0,4695 |
| Onsite light level: Shade | 0,0000 | 1,0000 |
| Onsite light level: Sunny | 0,0135 | 0,4935 |
| Onsite light level: Half-shade | 0,0201 | 0,3516 |
| Onsite moisture level: Fresh | 0,0000 | 1,0000 |
| **Onsite moisture level: Wet** | 0,0652 | **0,0290** |
| Onsite moisture level: Dry | 0,0289 | 0,2338 |
| Onsite moisture level: Damp | 0,0313 | 0,1918 |
| Exposition: W | 0,0234 | 0,2837 |
| Exposition: S | 0,0304 | 0,1838 |
| Exposition: SO | 0,0133 | 0,3497 |
| Exposition: N | 0,0352 | 0,1419 |
| Exposition: NW | 0,0025 | 0,8931 |
| Exposition: SW | 0,0236 | 0,2627 |
| Exposition: O | 0,0521 | 0,0559 |
| **Species** | 1,0000 | **0,0000** |
| **Season** | 0,3544 | **0,0010** |

***Quality Control***

The mzQuality workflow revealed a relative standard deviation (RSD) for the maximum RT of 1.97 s and a mz deviation of 1.67*10^3^ of all detected features in the 18 QC samples. These values are well within the expected range of our analytical setup [94].

**Figure S7**: Quality control as employed by the mzQuality workflow. The plot shows the batch design (QC samples in light grey and the samples in dark grey) and the intensity measured in 22 metabolites in all measured samples.

***Raw chromatograms grouped by seasons***

In order to assess the quality of the mass spectrometry runs, following are plots of raw chromatograms which are grouped by seasons.

**Figure S8**: Total ion chromatograms for the nine tested species grouped by seasons.
