# Supplementary figures and images for: Chemical Diversity and Classification of Secondary Metabolites in Nine Bryophyte Species
Source: Metabolites. 2019 Oct 11;9(10):222. doi: 10.3390/metabo9100222 (PMC6835487; doi:10.3390/metabo9100222)

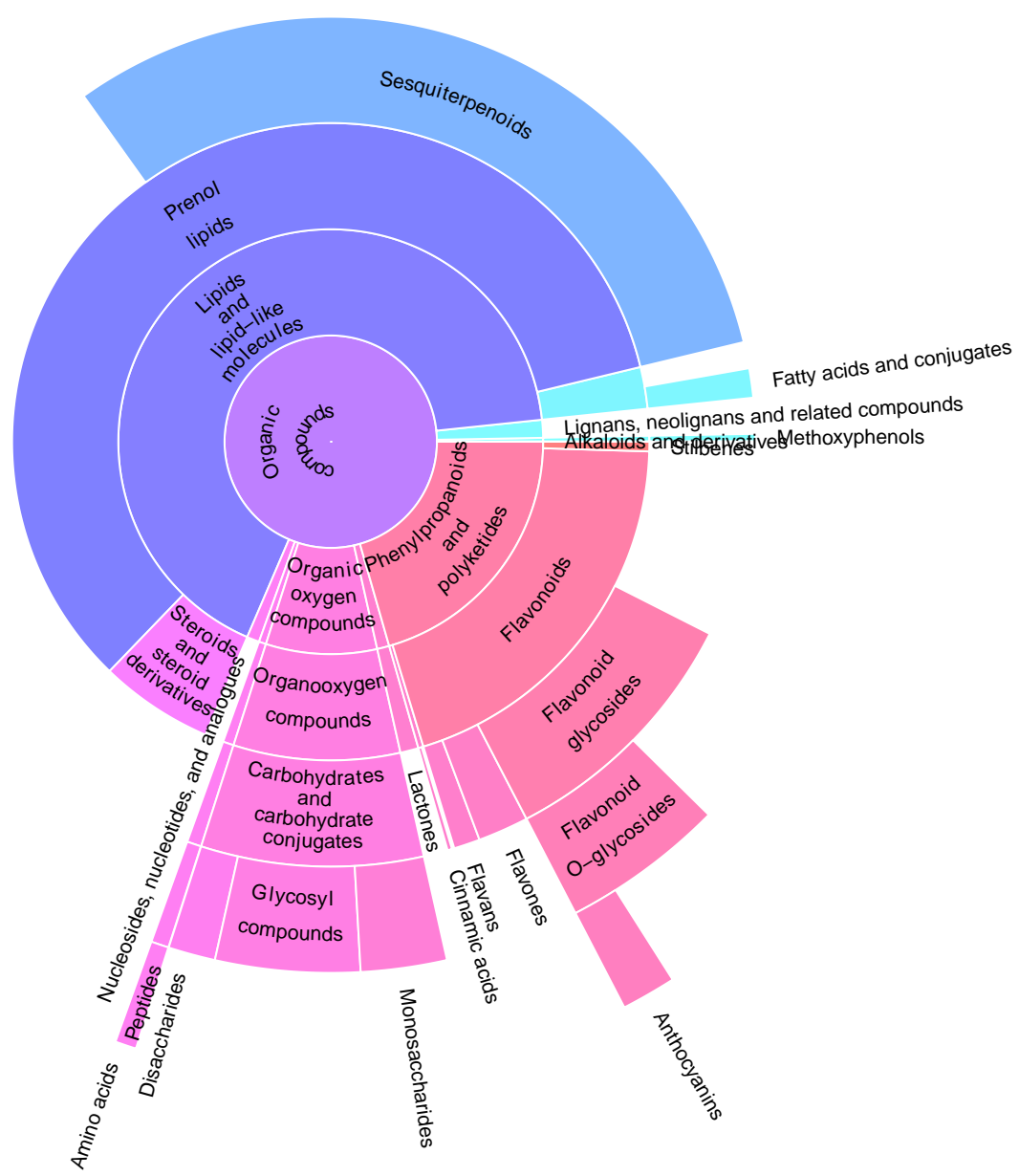

Supplement: Supplementary file 1 [file metabolites-09-00222-s001.zip › fig_1.pdf]

(a)

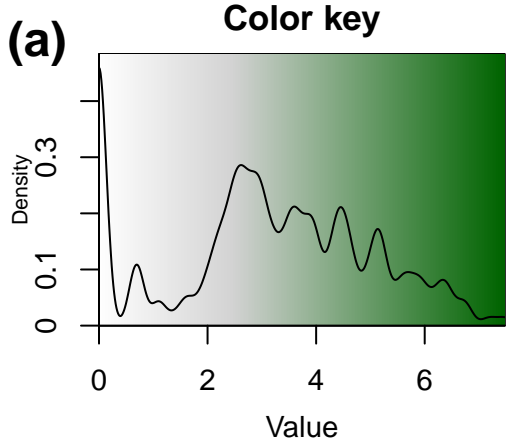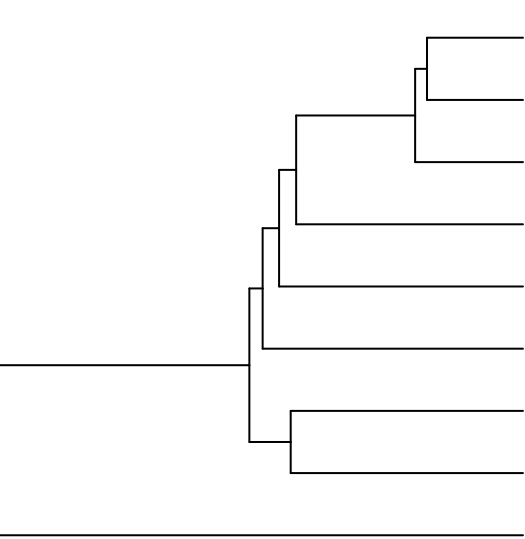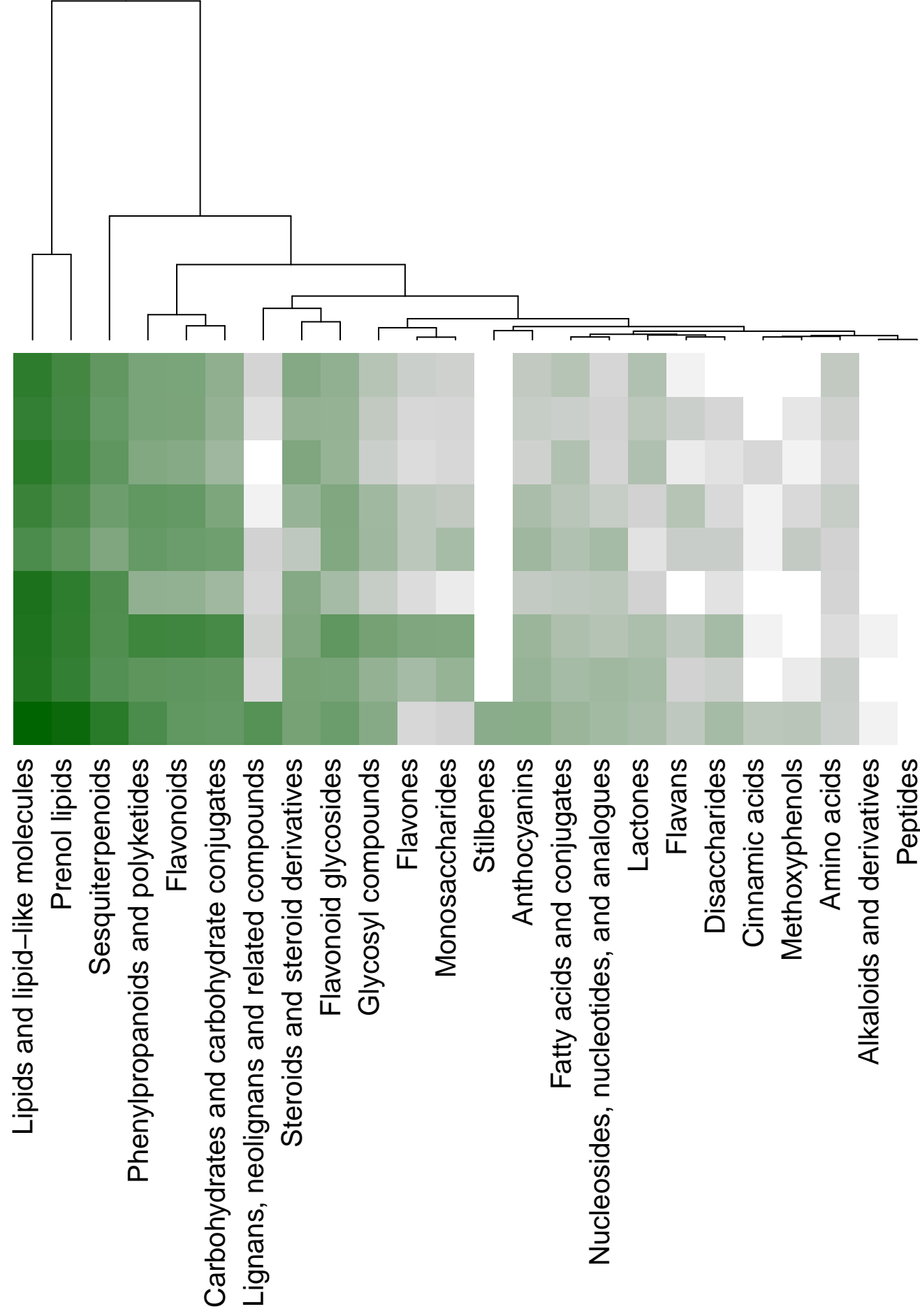

Hypcup

Calcus

Brarut

Rhysqu

Polstr

Gripul

Plaund

Fistax

Marpol

(b)

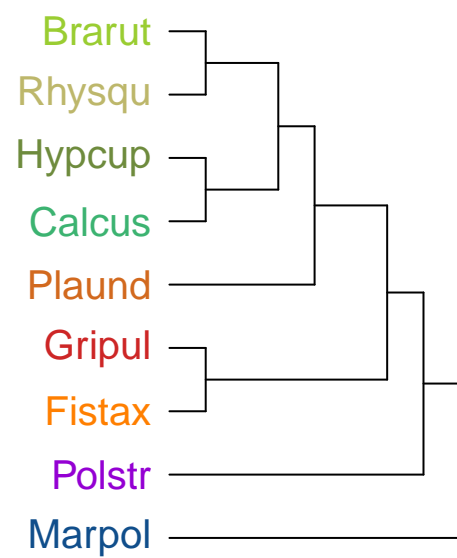

Supplement: Supplementary file 1 [file metabolites-09-00222-s001.zip › fig_2.pdf]

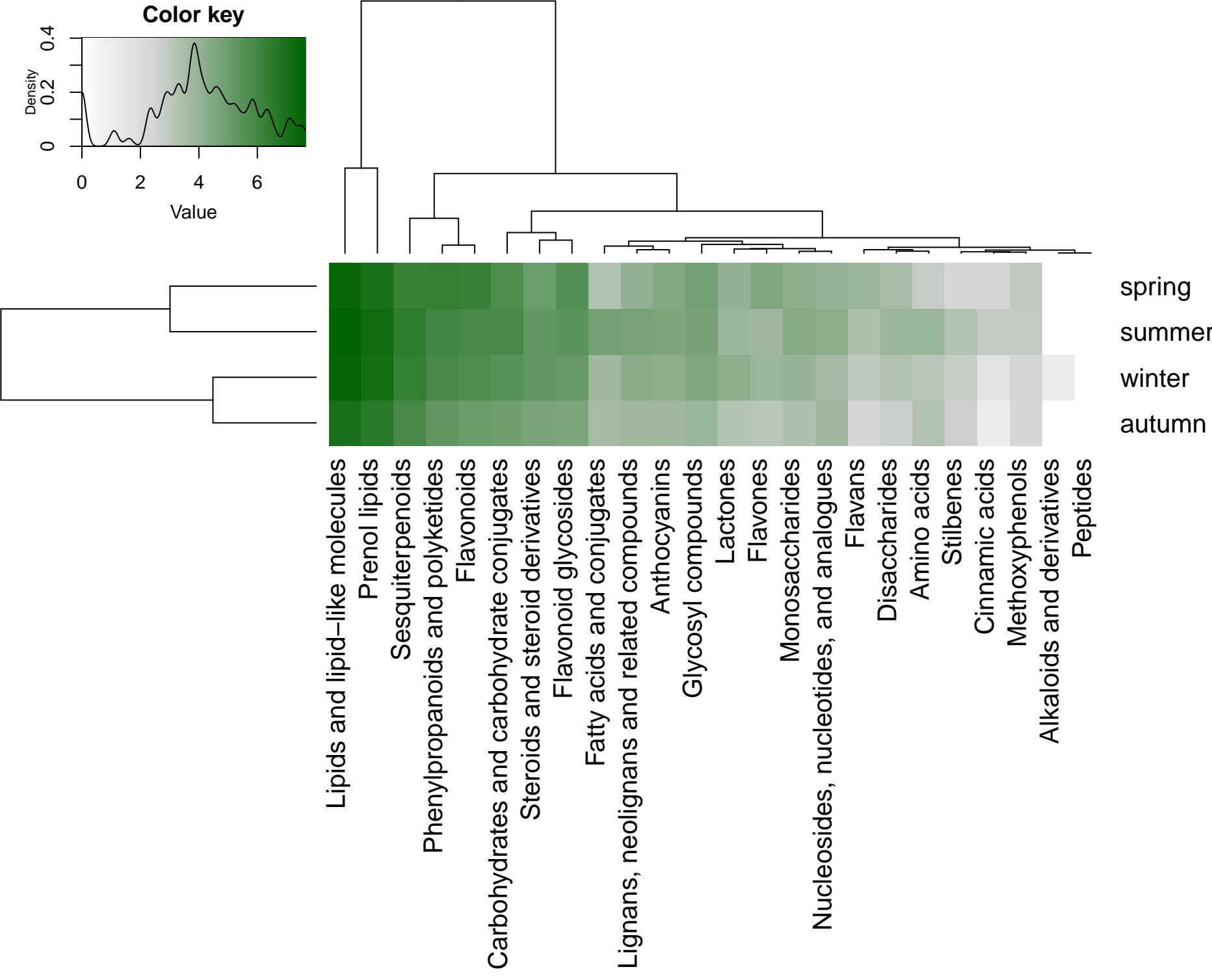

Supplement: Supplementary file 1 [file metabolites-09-00222-s001.zip › fig_4.pdf]

# Number of unique features

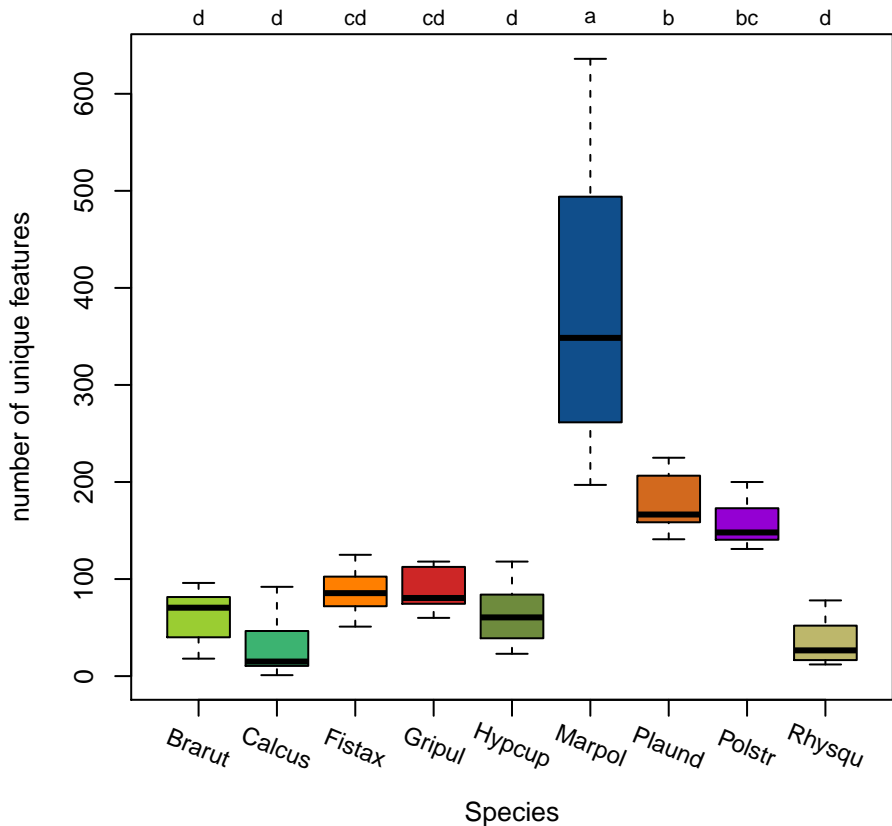

Supplement: Supplementary file 1 [file metabolites-09-00222-s001.zip › fig_s1a.pdf]

# Shannon diversity ( $H'$ )

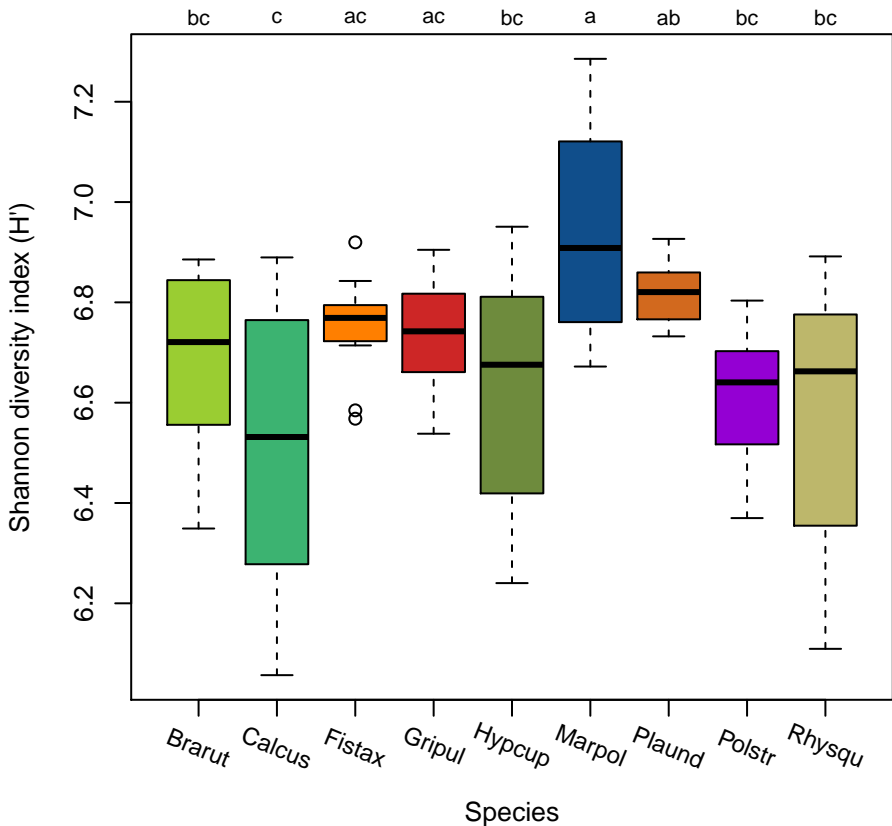

Supplement: Supplementary file 1 [file metabolites-09-00222-s001.zip › fig_s1b.pdf]

## Pielou's evenness

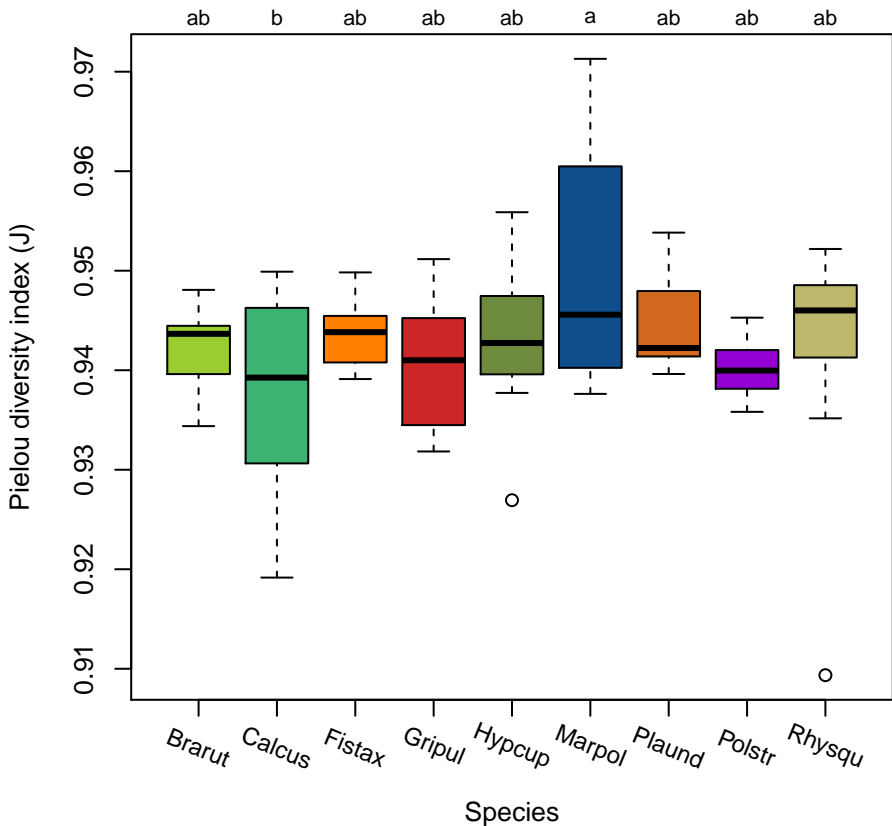

Supplement: Supplementary file 1 [file metabolites-09-00222-s001.zip › fig_s1c.pdf]

# Concentration

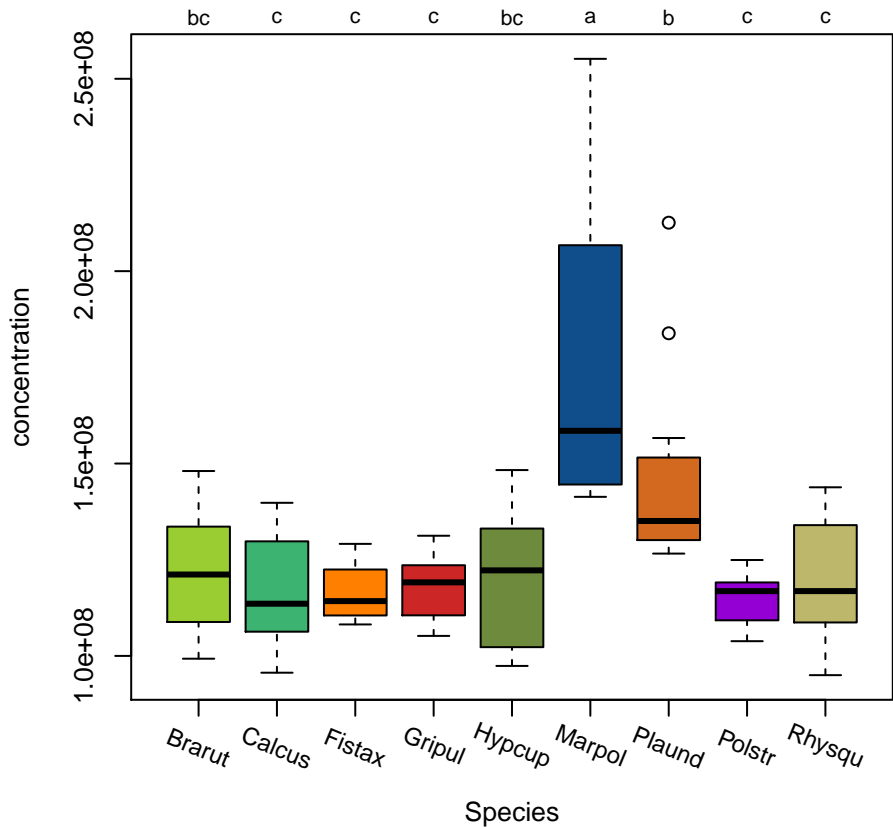

Supplement: Supplementary file 1 [file metabolites-09-00222-s001.zip › fig_s1d.pdf]

# Number of unique features

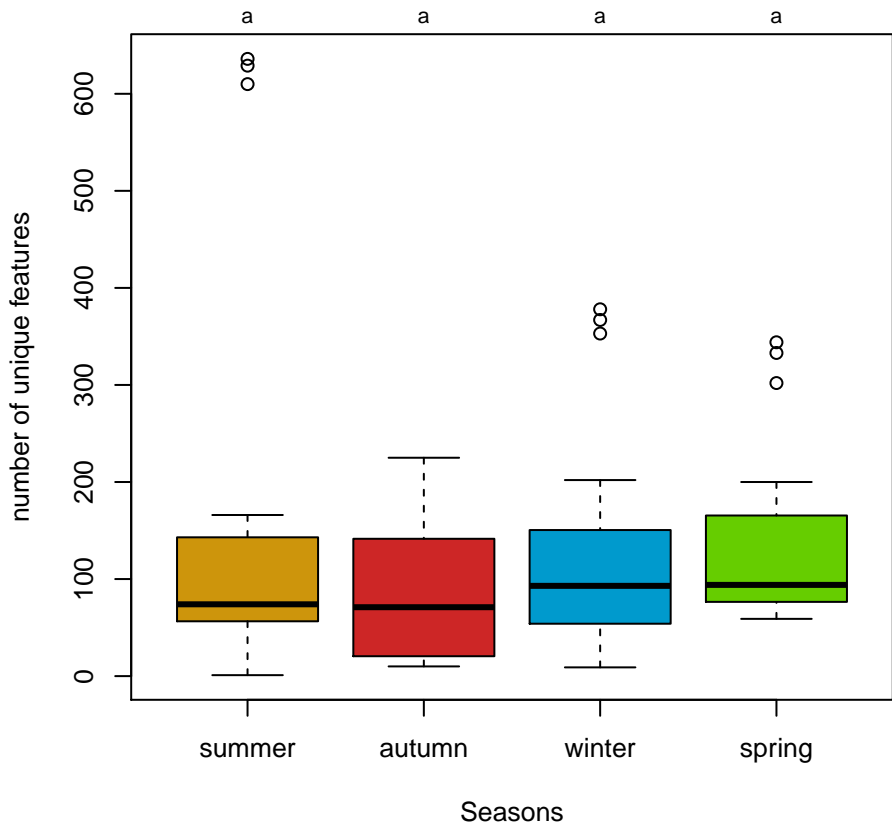

Supplement: Supplementary file 1 [file metabolites-09-00222-s001.zip › fig_s2a.pdf]

# Shannon diversity (H')

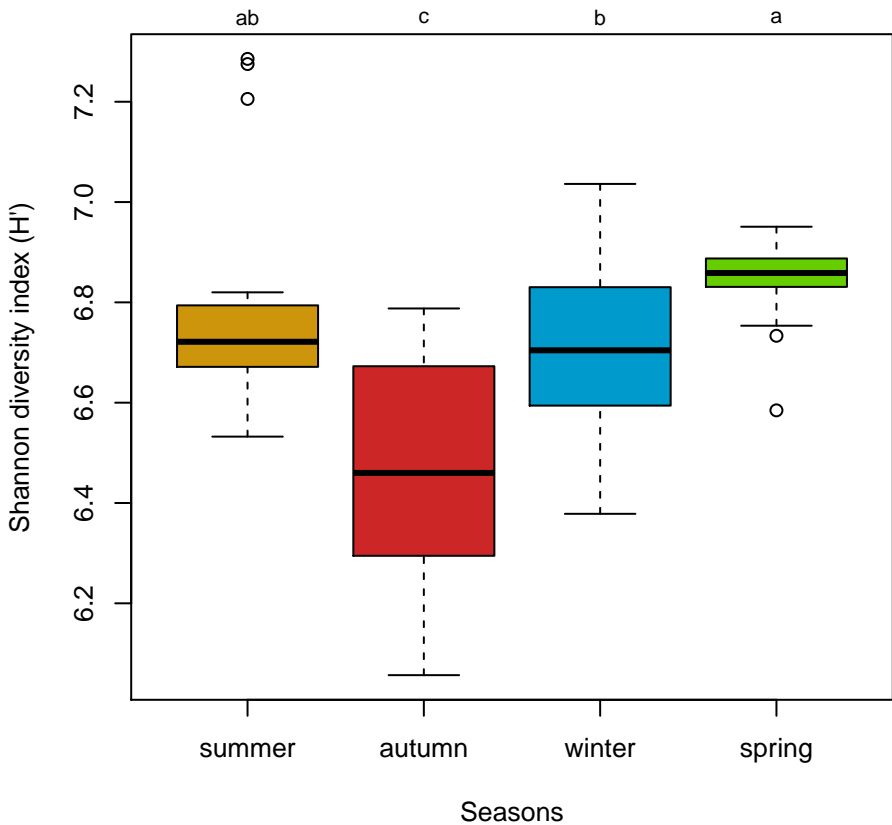

Supplement: Supplementary file 1 [file metabolites-09-00222-s001.zip › fig_s2b.pdf]

## Pielou's evenness

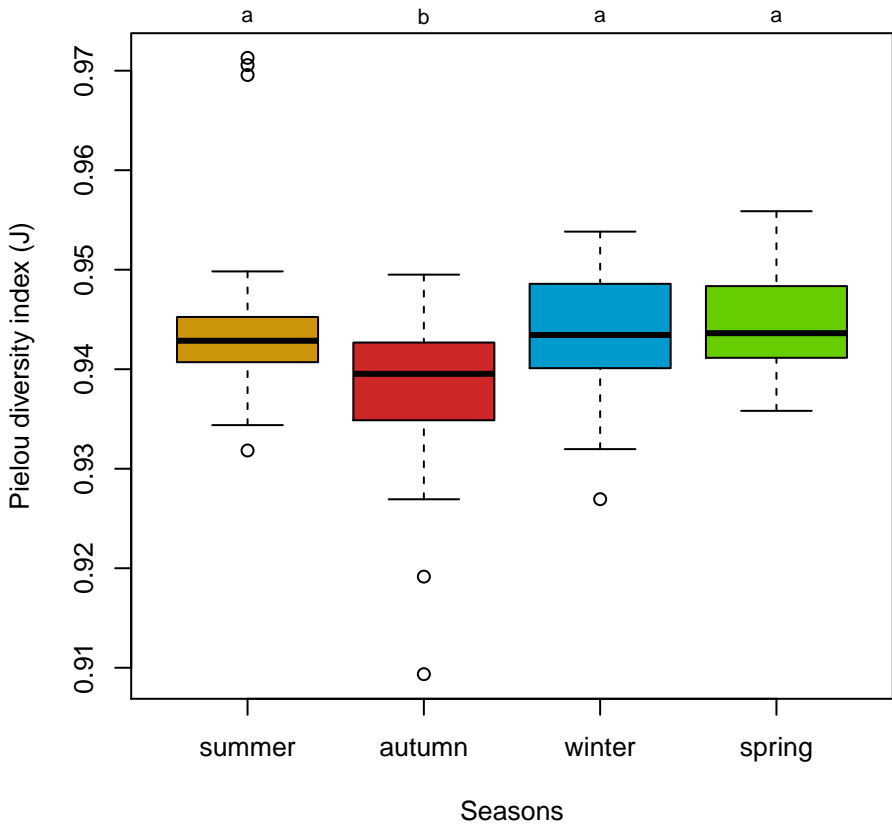

Supplement: Supplementary file 1 [file metabolites-09-00222-s001.zip › fig_s2c.pdf]

# Concentration

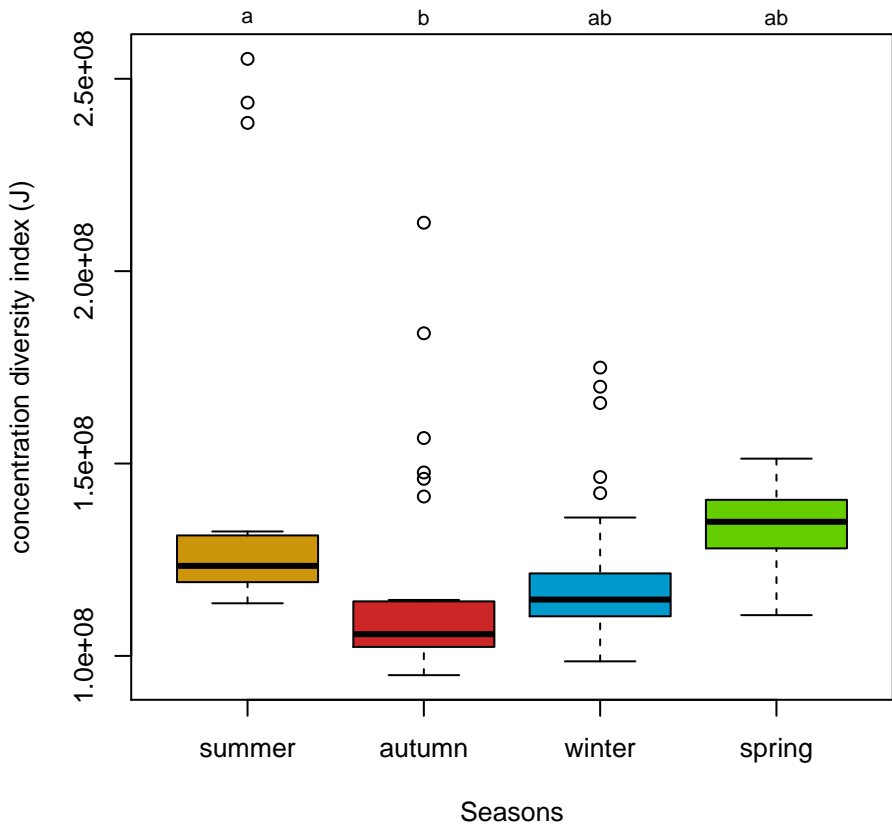

Supplement: Supplementary file 1 [file metabolites-09-00222-s001.zip › fig_s2d.pdf]

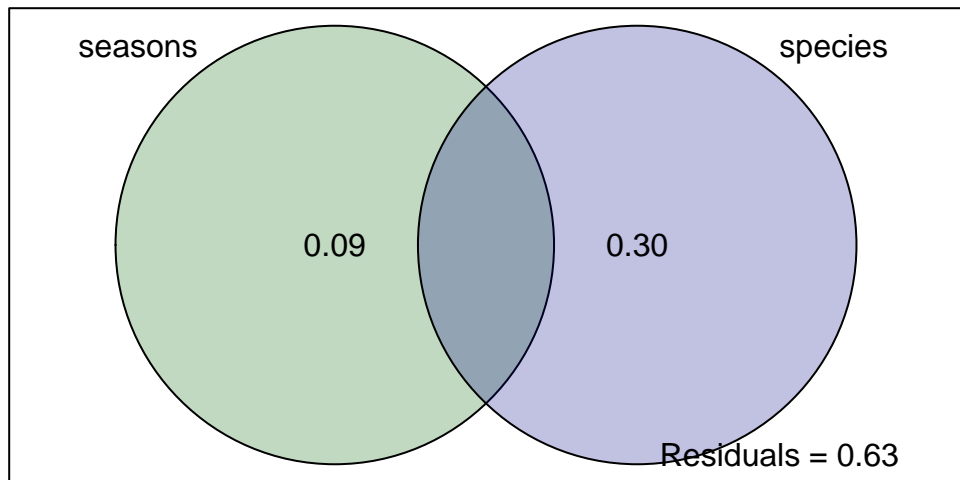

Values <0 not shown

Supplement: Supplementary file 1 [file metabolites-09-00222-s001.zip › fig_s3a.pdf]

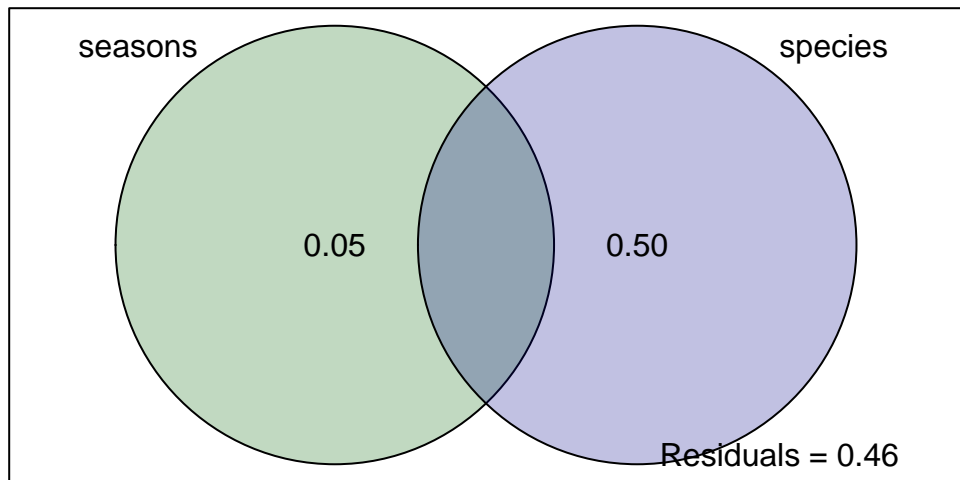

Values <0 not shown

Supplement: Supplementary file 1 [file metabolites-09-00222-s001.zip › fig_s3b.pdf]

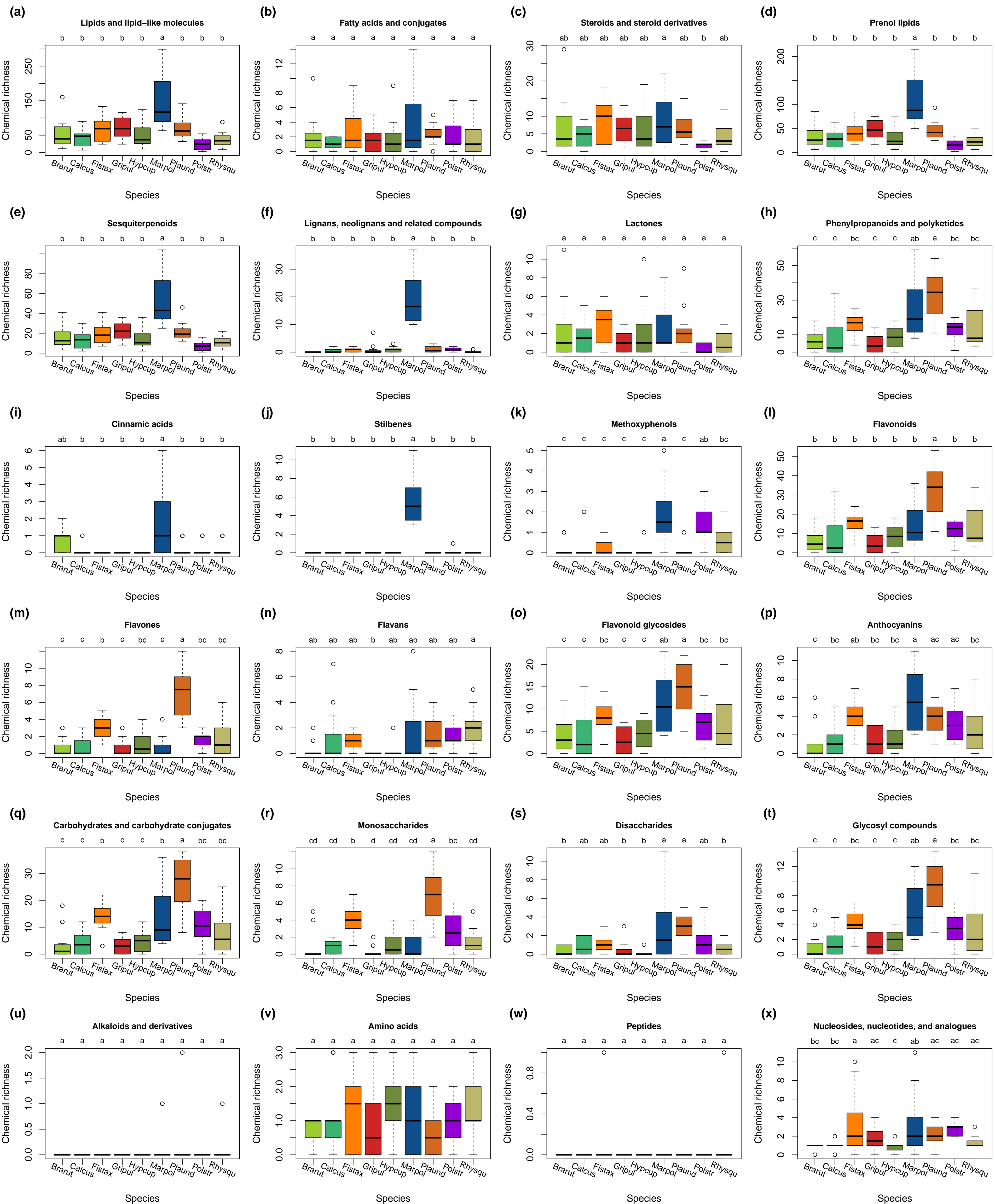

Supplement: Supplementary file 1 [file metabolites-09-00222-s001.zip › fig_s4.pdf]

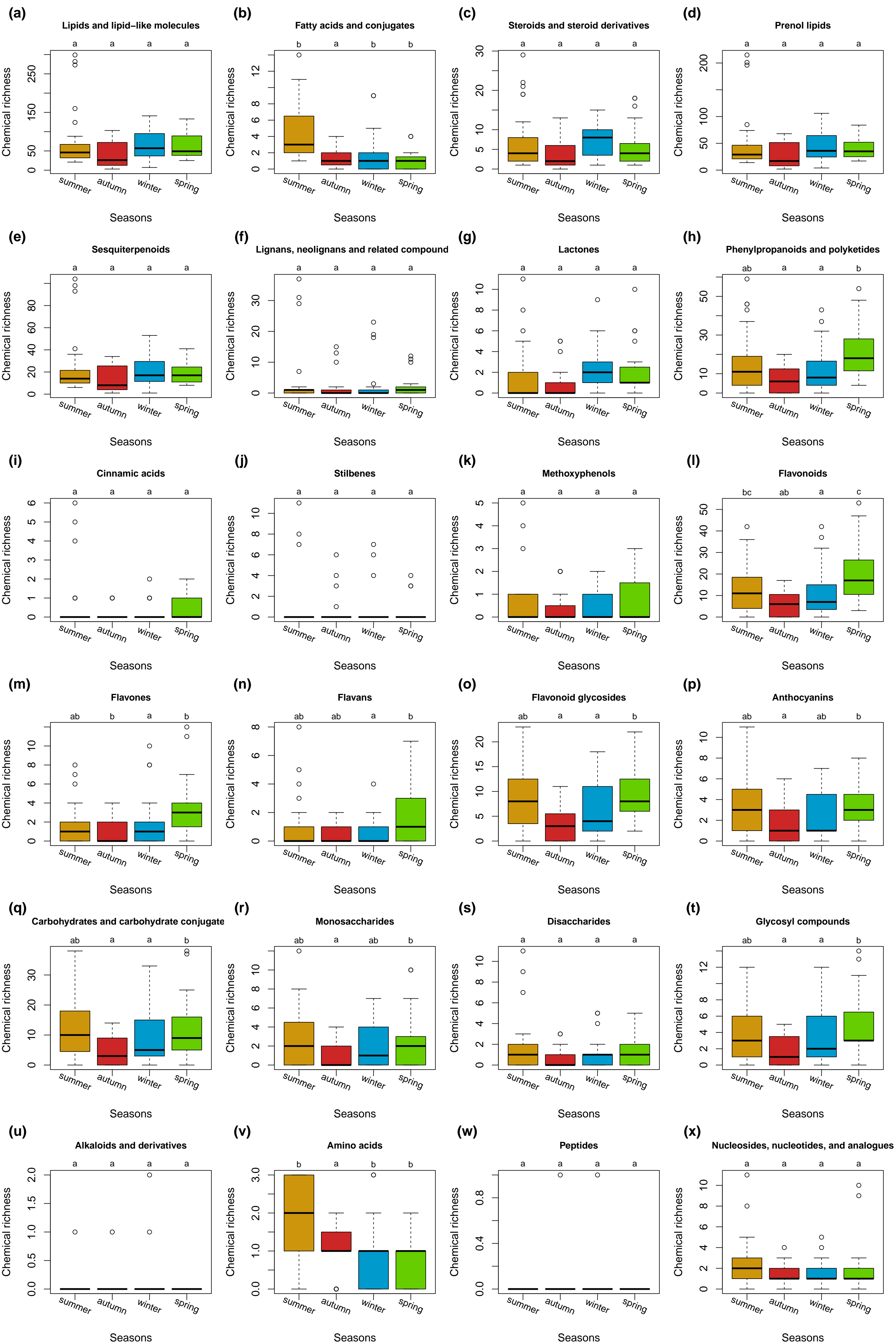

Supplement: Supplementary file 1 [file metabolites-09-00222-s001.zip › fig_s5.pdf]
