# Supplementary material for: Chemical Diversity and Classification of Secondary Metabolites in Nine Bryophyte Species
Source: Metabolites. 2019 Oct 11;9(10):222. doi: 10.3390/metabo9100222 (PMC6835487; doi:10.3390/metabo9100222)

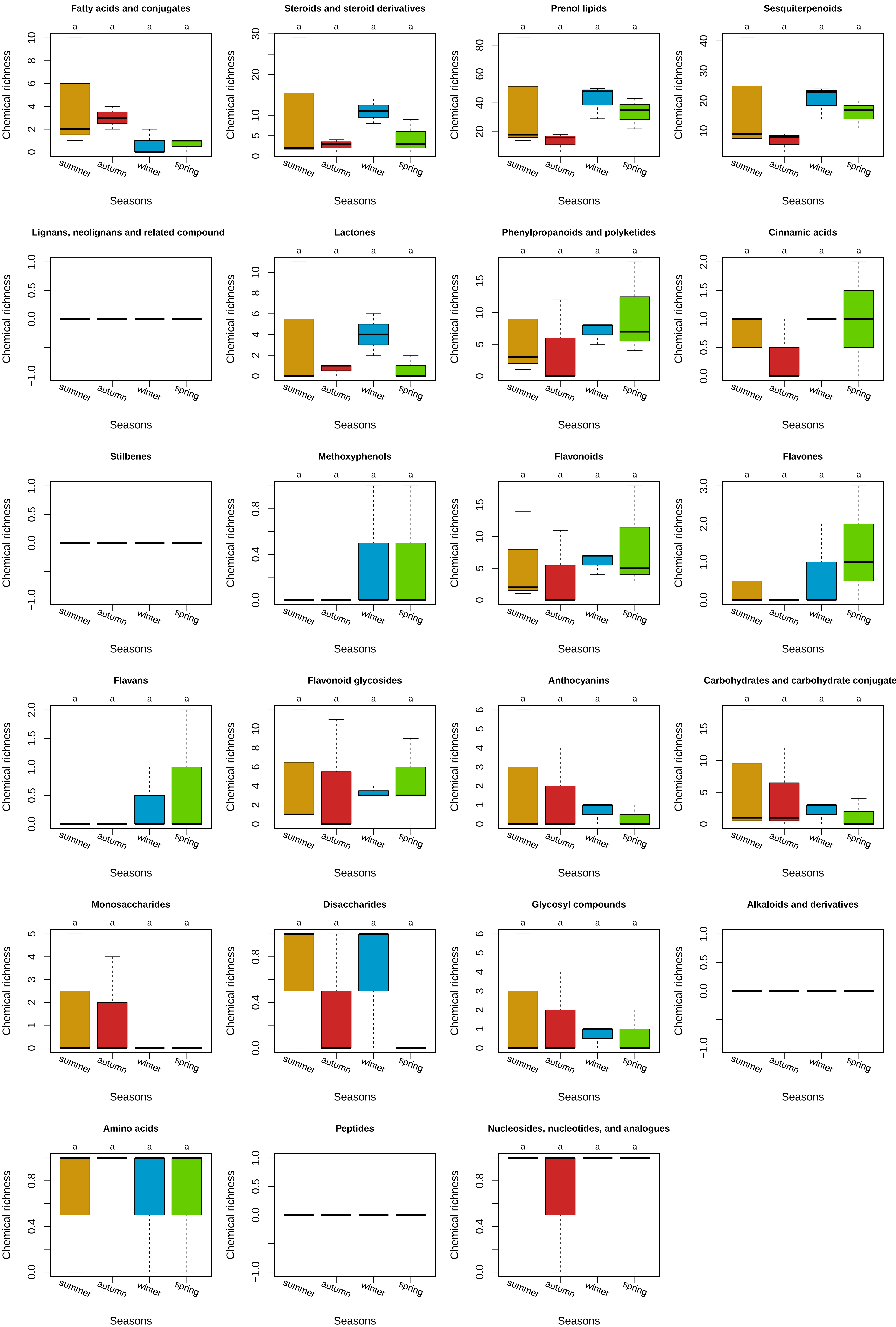
Fatty acids and conjugates
Steroids and steroid derivatives
Prenol lipids
Sesquiterpenoids
Lignans, neolignans and related compound
Lactones
Phenylpropanoids and polyketides
Cinnamic acids
Stilbenes
Methoxyphenols
Flavonoids
Flavones
Flavans
Flavonoid glycosides
Anthocyanins
Carbohydrates and carbohydrate conjugate
Monosaccharides
Disaccharides
Glycosyl compounds
Alkaloids and derivatives
Amino acids
Peptides
Nucleosides, nucleotides, and analogues
Chemical richness
Seasons
summer
autumn
winter
spring
a

(c) Fissidens taxifolius

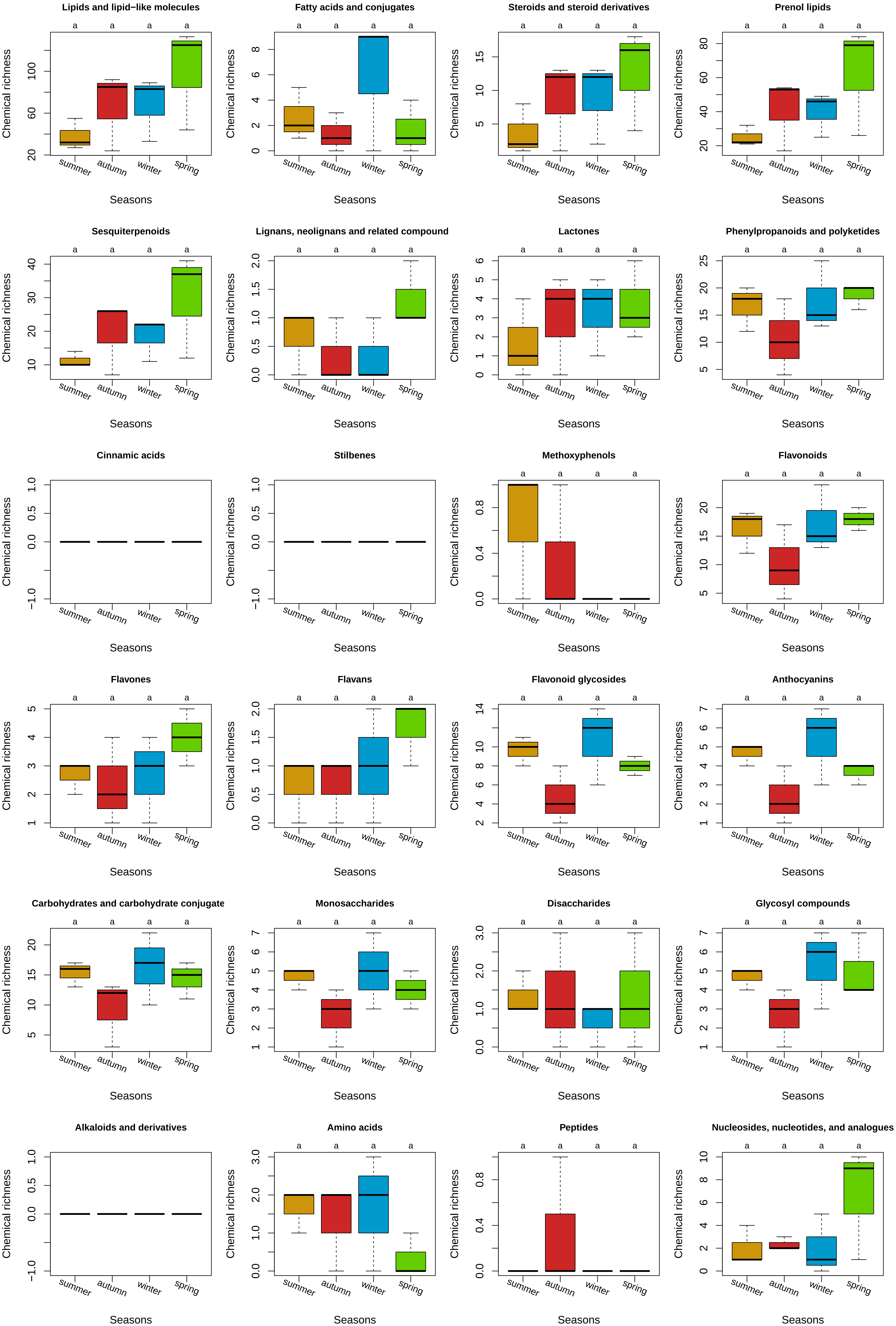

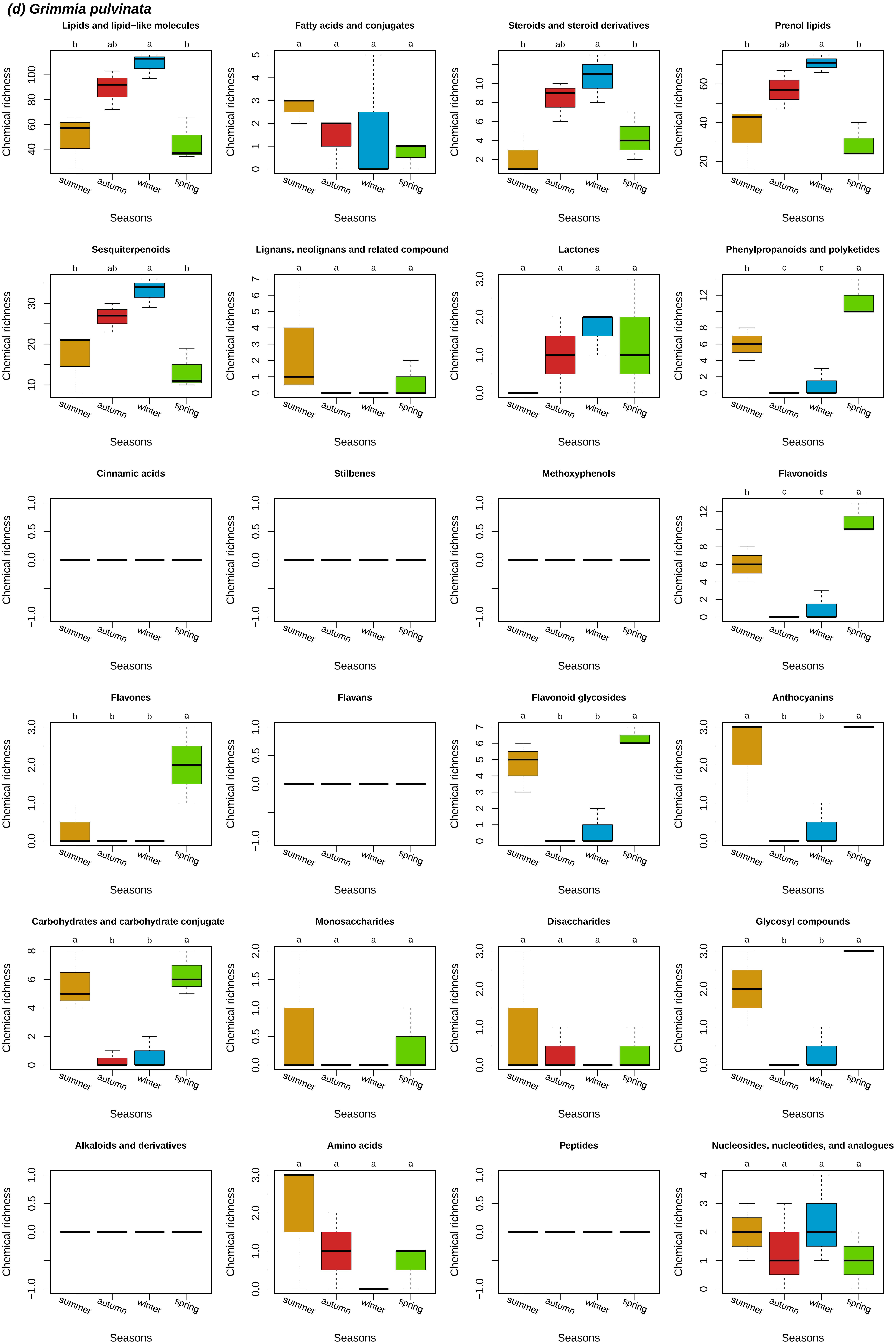
(d) Grimmia pulvinata
Lipids and lipid−like molecules
b ab a b
Fatty acids and conjugates
a a a a
Steroids and steroid derivatives
b ab a b
Prenol lipids
b ab a b
Sesquiterpenoids
b ab a b
Lignans, neolignans and related compound
a a a a
Lactones
a a a a
Phenylpropanoids and polyketides
b c c a
Cinnamic acids
Stilbenes
Methoxyphenols
Flavonoids
b c c a
Flavones
b b b a
Flavans
Flavonoid glycosides
a b b a
Anthocyanins
a b b a
Carbohydrates and carbohydrate conjugate
a b b a
Monosaccharides
a a a a
Disaccharides
a a a a
Glycosyl compounds
a b b a
Alkaloids and derivatives
Amino acids
a a a a
Peptides
Nucleosides, nucleotides, and analogues
a a a a
Chemical richness
summer
autumn
winter
spring
Seasons

(e) Hypnum cupressiforme

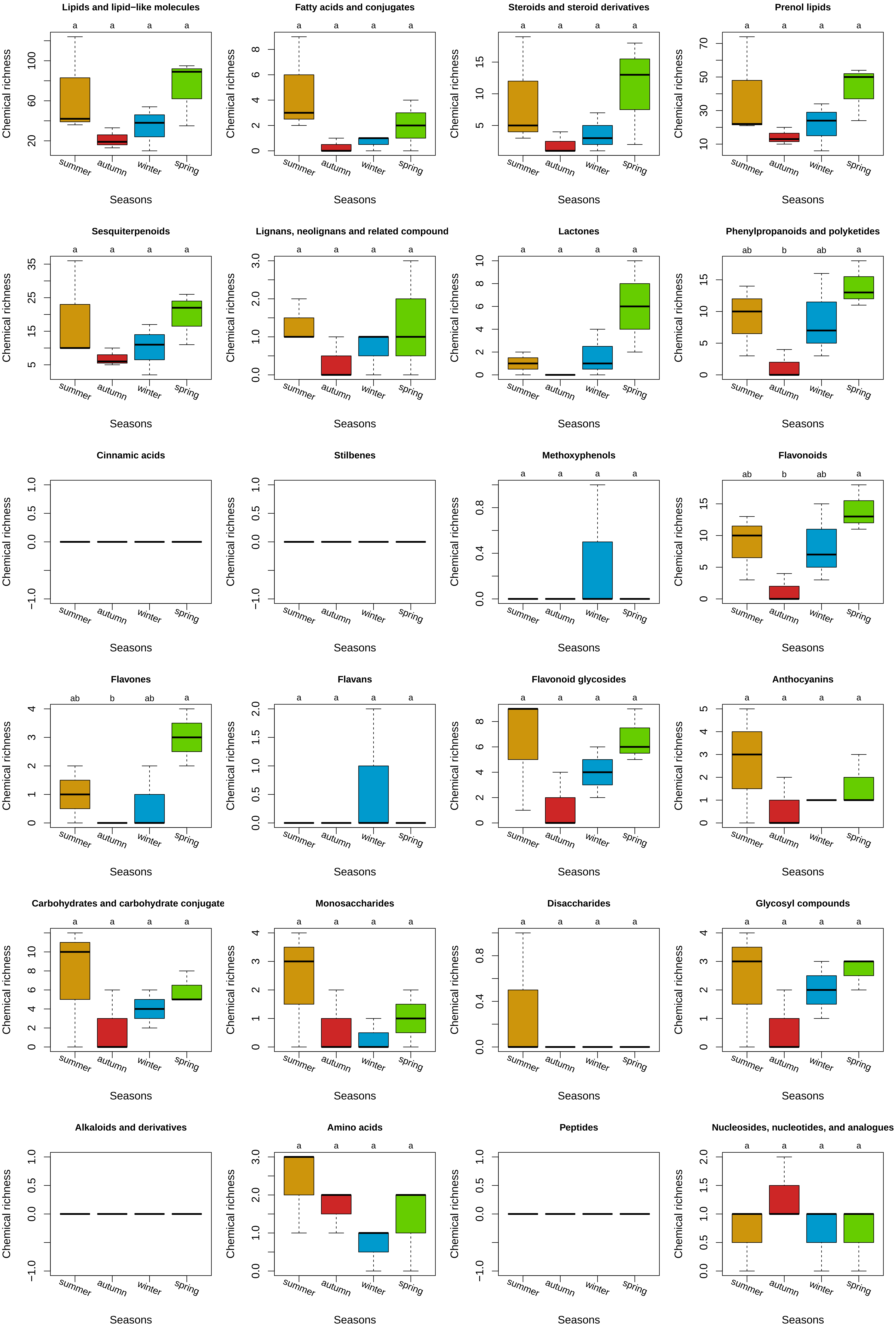

(f) *Marchantia polymorpha*

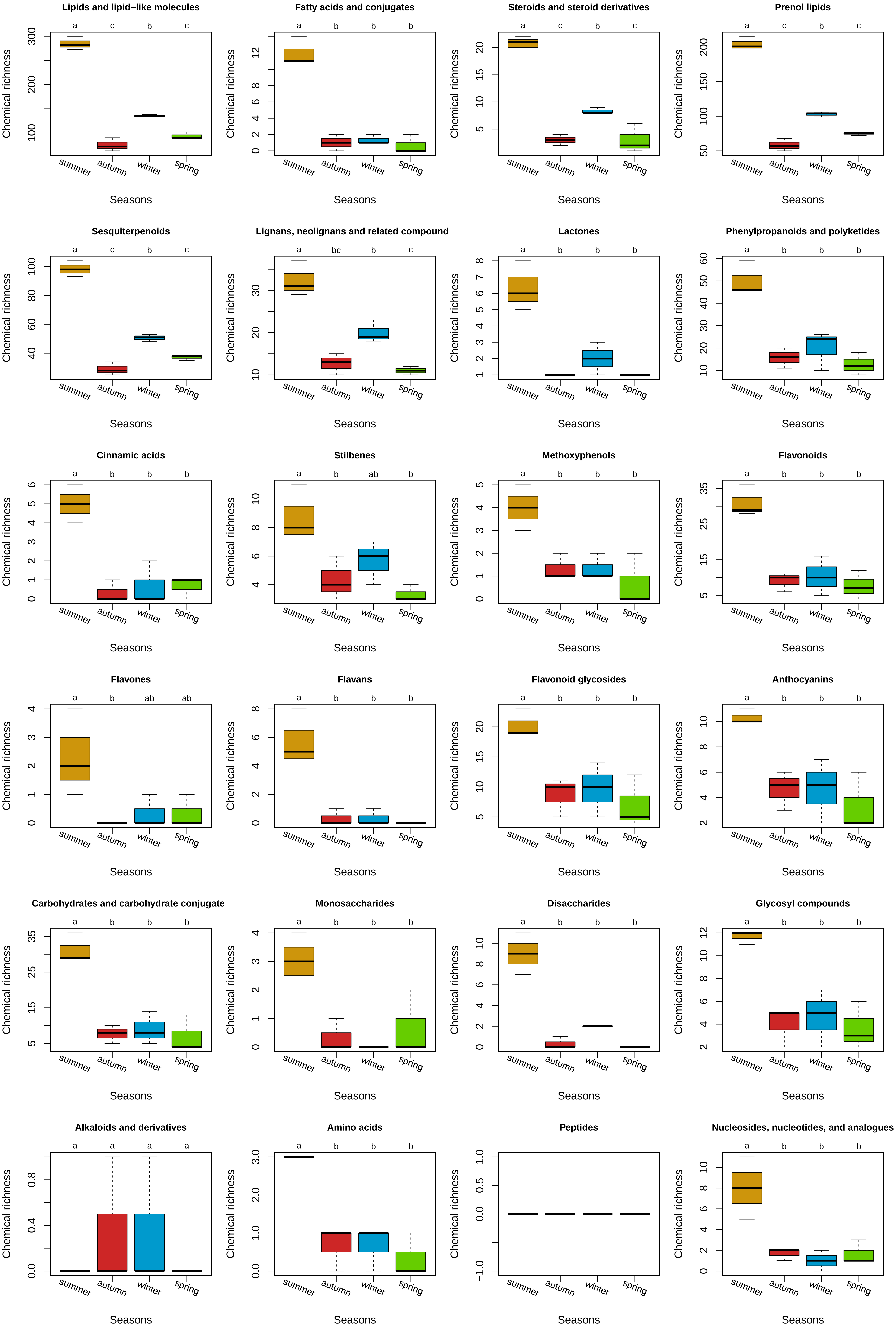

(g) *Plagiomnium undulatum*

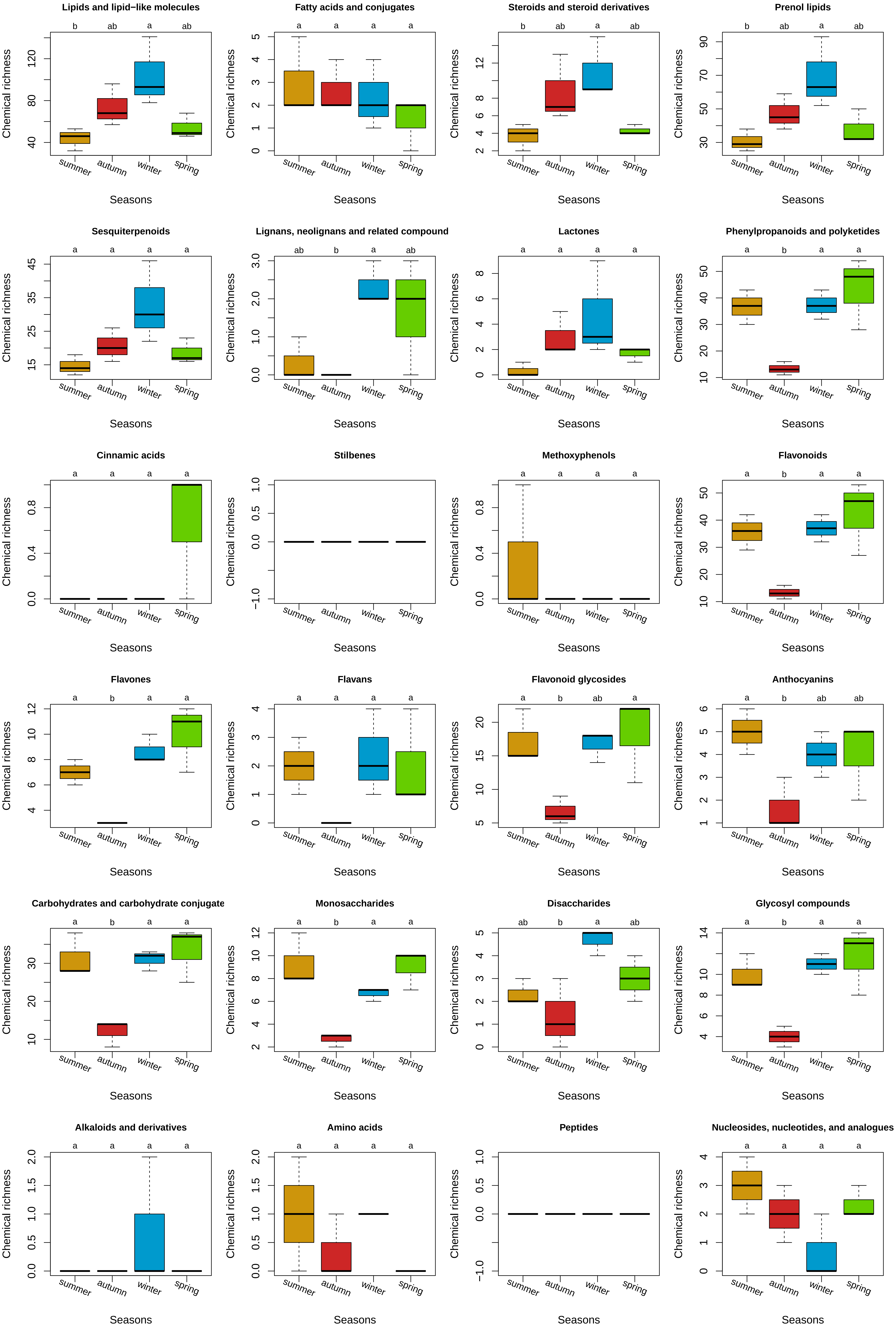

(h) Polytrichum strictum

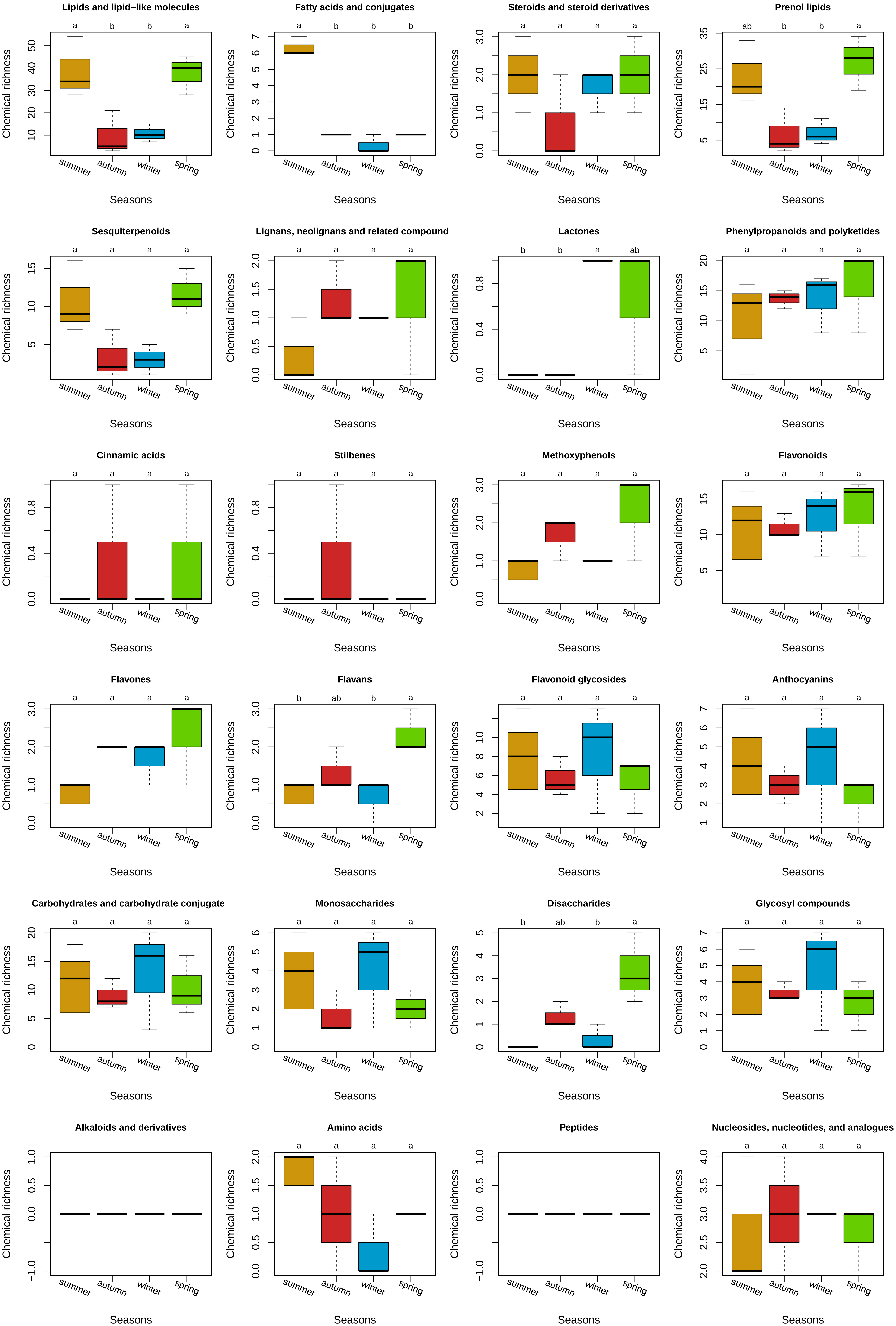

(i) Rhytidiadelphus squarrosus

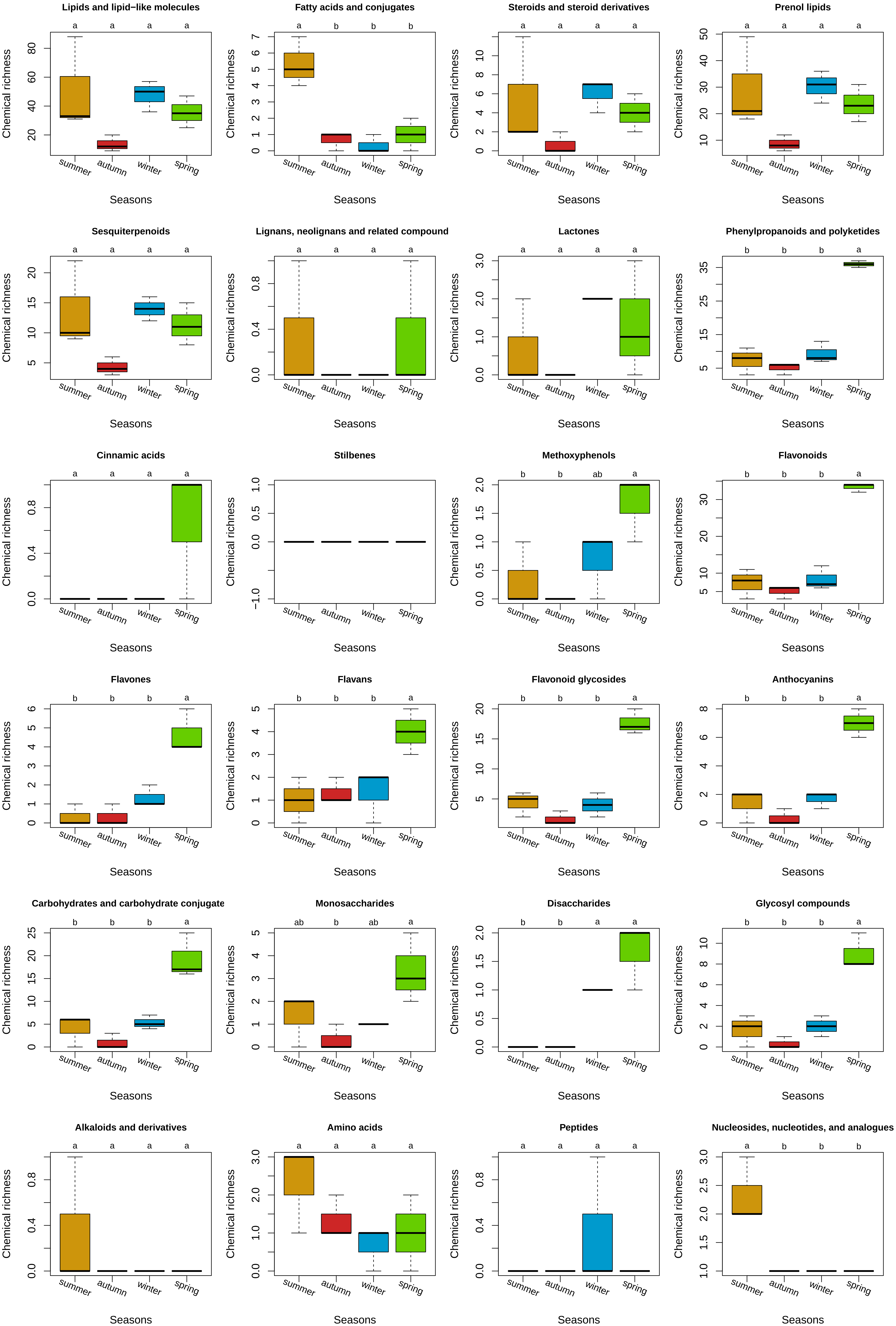

(j) Pleurocarpous species

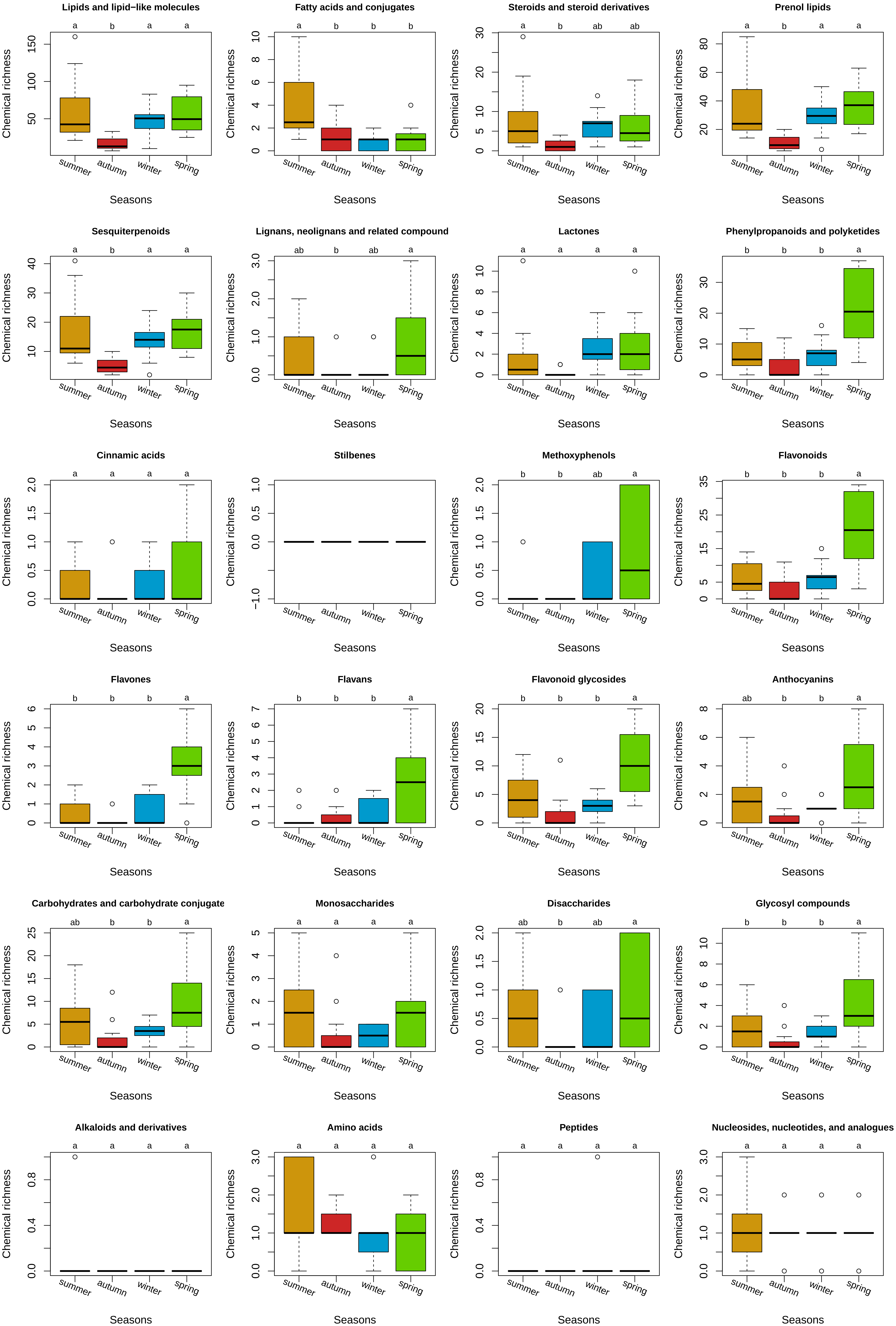

(k) Acrocarpous species

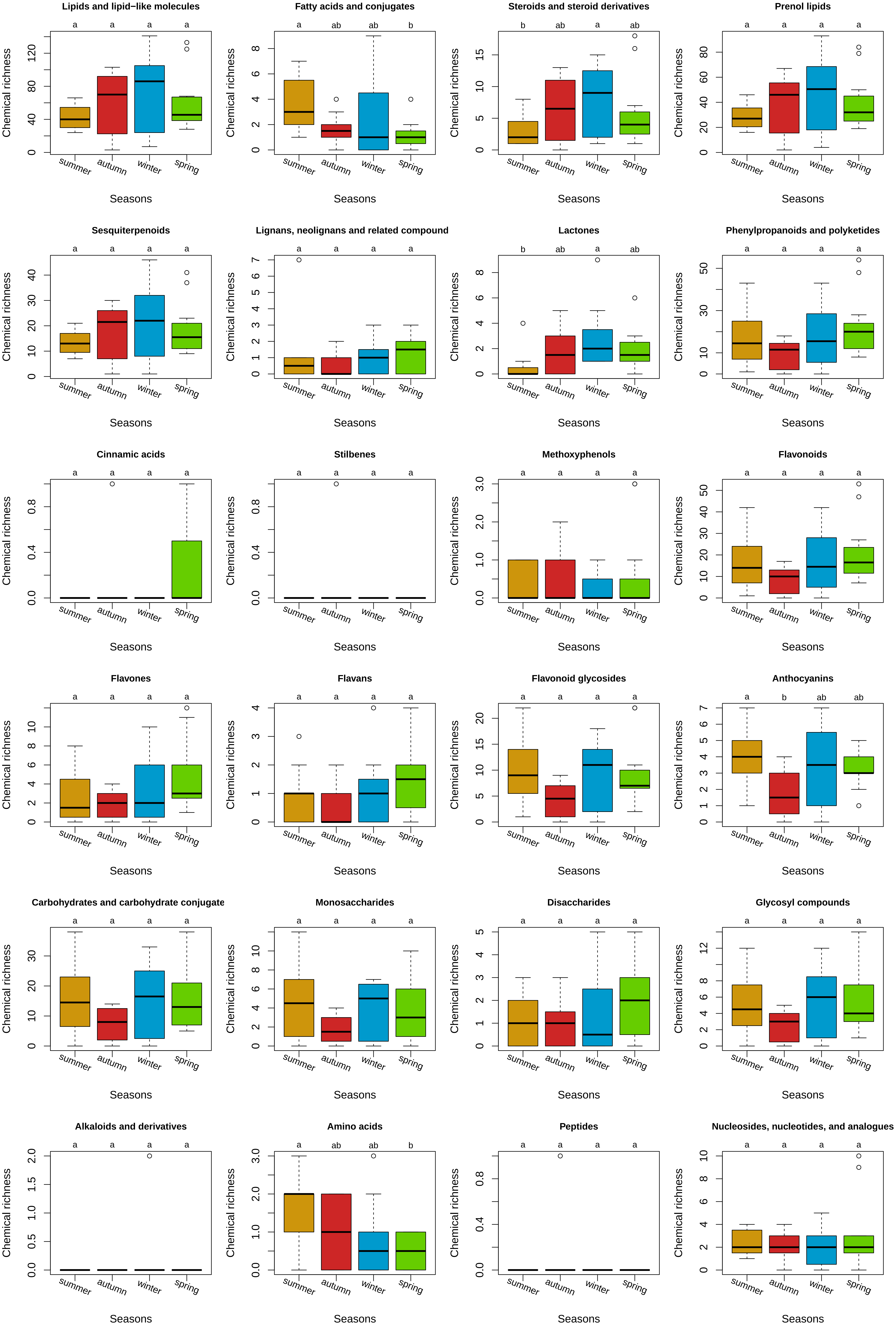

Supplement: Supplementary file 1 [file fig_s6.pdf › fig_s6.pdf]
